# Elongation Factor Tu Prevents Misediting of Gly-tRNA(Gly) Caused by the Design Behind the Chiral Proofreading Site of D-Aminoacyl-tRNA Deacylase

**DOI:** 10.1371/journal.pbio.1002465

**Published:** 2016-05-25

**Authors:** Satya Brata Routh, Komal Ishwar Pawar, Sadeem Ahmad, Swati Singh, Katta Suma, Mantu Kumar, Santosh Kumar Kuncha, Kranthikumar Yadav, Shobha P Kruparani, Rajan Sankaranarayanan

**Affiliations:** 1 CSIR–Centre for Cellular and Molecular Biology, Hyderabad, India; 2 CSIR–Indian Institute of Chemical Technology, Hyderabad, India; Ohio State University, UNITED STATES

## Abstract

D-aminoacyl-tRNA deacylase (DTD) removes D-amino acids mischarged on tRNAs and is thus implicated in enforcing homochirality in proteins. Previously, we proposed that selective capture of D-aminoacyl-tRNA by DTD’s invariant, cross-subunit Gly-*cis*Pro motif forms the mechanistic basis for its enantioselectivity. We now show, using nuclear magnetic resonance (NMR) spectroscopy-based binding studies followed by biochemical assays with both bacterial and eukaryotic systems, that DTD effectively misedits Gly-tRNA^Gly^. High-resolution crystal structure reveals that the architecture of DTD’s chiral proofreading site is completely porous to achiral glycine. Hence, L-chiral rejection is the only design principle on which DTD functions, unlike other chiral-specific enzymes such as D-amino acid oxidases, which are specific for D-enantiomers. Competition assays with elongation factor thermo unstable (EF-Tu) and DTD demonstrate that EF-Tu precludes Gly-tRNA^Gly^ misediting at normal cellular concentrations. However, even slightly higher DTD levels overcome this protection conferred by EF-Tu, thus resulting in significant depletion of Gly-tRNA^Gly^. Our in vitro observations are substantiated by cell-based studies in *Escherichia coli* that show that overexpression of DTD causes cellular toxicity, which is largely rescued upon glycine supplementation. Furthermore, we provide direct evidence that DTD is an RNA-based catalyst, since it uses only the terminal 2′-OH of tRNA for catalysis without the involvement of protein side chains. The study therefore provides a unique paradigm of enzyme action for substrate selection/specificity by DTD, and thus explains the underlying cause of DTD’s activity on Gly-tRNA^Gly^. It also gives a molecular and functional basis for the necessity and the observed tight regulation of DTD levels, thereby preventing cellular toxicity due to misediting.

## Abstract 2

The enzyme D-aminoacyl-tRNA deacylase (DTD) proofreads acyl tRNAs to remove D-amino acids. Mechanistic insights into DTD reveal that it can “mis-edit” the achiral amino acid glycine on its tRNA, but that this is prevented by elongation factor Tu.

## Author Summary

Proteins are composed of 20 different amino acids, 19 of which have L-chirality and one, glycine, that is achiral. A polypeptide consisting of both D- and L-amino acids cannot assume its proper three-dimensional conformation and is therefore non-functional. Since the cellular milieu contains both D- and L-amino acids, the protein synthesis machinery employs molecules to prevent D-amino acid incorporation into proteins. One key molecule in this process is an enzyme named D-aminoacyl-tRNA deacylase (DTD), which hydrolyses D-amino acids attached to tRNA molecules (a D-aminoacyl-tRNA). Here, we show that besides acting on D-aminoacyl-tRNA, DTD also efficiently hydrolyses the achiral glycine attached to tRNA—glycyl-tRNA(Gly)—as DTD’s active site pocket cannot discriminate between D-amino acids and glycine. Thus, DTD is designed to reject substrates with L-chirality, rather than target substrates with D-chirality. DTD’s activity on glycyl-tRNA(Gly) is potentially harmful for the cell because glycine is an essential constituent of proteins. We further demonstrate that elongation factor thermo unstable (EF-Tu), required for delivering aminoacyl-tRNAs to the ribosome for protein synthesis, protects glycyl-tRNA(Gly) against DTD, thereby preventing DTD-induced cellular toxicity. We suggest that DTD levels in the cell must be tightly regulated to maintain cellular homeostasis.

## Introduction

Proofreading at various steps of translation of the genetic code—DNA replication, transcription, and translation—ensures that errors are kept at such low levels that they help the cell to evolve without jeopardizing cell viability. It is a multi-factorial process in which different molecules, including the ribosome [1], work in tandem to maintain fidelity of transfer of genetic information. One such process at the level of protein synthesis involves aminoacyl-tRNA synthetases (aaRSs), which charge amino acids on their cognate tRNAs [2]. About half of the 20 usually occurring aaRSs mischarge non-cognate L-amino acids, which are chemically or structurally similar to their cognate counterparts. For instance, threonyl-tRNA synthetase (ThrRS) mischarges non-cognate L-serine, which differs from the cognate L-threonine by just a methyl group. These aaRSs that face such a subtle discrimination problem are provided with a dedicated proofreading/editing domain that removes the non-cognate L-amino acid from the tRNA [3]. Mutations causing defects in these proofreading mechanisms have been shown to result in adverse functional consequences in various systems from bacteria to mammalian cells, including neurodegeneration in mice [4–6].

Some aaRSs, such as tyrosyl-tRNA synthetase, commit “chiral errors” by mischarging the D-enantiomer of the amino acid on tRNA [7]. This kind of error can be magnified in certain tissues in which some D-amino acids are present at high levels. For example, in neuronal tissues, D-serine and D-glutamate, which act as neurotransmitters, are found in abundance [8–10]. D-amino acids mischarged on tRNAs might get incorporated into polypeptides [11] and form heterochiral proteins, which cannot assume proper conformation leading to misfolding; or they can deplete the free tRNA pool required for the delivery of L-amino acids to the ribosome for protein synthesis [12]. Either scenario is detrimental to the cell and is efficiently avoided by deploying the enzyme D-aminoacyl-tRNA deacylase (DTD), which decouples D-amino acids erroneously charged on tRNAs [11,13,14]. The proofreading activity of DTD, present ubiquitously across bacteria and eukaryotes, has been termed as “chiral proofreading” [15]. Thus, DTD works as the major chiral checkpoint—apart from others, including the ribosome [16]—that prevents infiltration of D-amino acids into the translational machinery, thereby enforcing homochirality in proteins.

Proofreading mechanisms of many aaRSs dealing with non-cognate L-amino acids have been studied extensively from both biochemical and structural perspectives [17–28]. However, mechanistically, little was known about how the cell excludes D-amino acids from the translational apparatus, although DTD was discovered in 1967 and the first structure of the enzyme was reported in 2001 [11,13,14,29–33]. In our recent work, we deciphered the structural basis for DTD’s enantioselectivity. An invariant, cross-subunit Gly-*cis*Pro motif in DTD, inserted into the active site of the dimeric counterpart, is responsible for selectively capturing the chiral centre of D-aminoacyl-tRNA [15].

Whether DTD achieves its remarkable enantioselectivity through D-chiral selection, L-chiral rejection, or a combination of both was a critical aspect that needed to be addressed. Therefore, in this work we show that L-chiral rejection is the only design principle on which DTD functions, and the D-amino acid binding mode is just happenstance, as it is the only possible mode in which the D-enantiomer can be accommodated in the pocket. Consequently, the chiral proofreading site can reject even the smallest L-chiral substrate, i.e., L-alanine, but cannot discriminate between D-amino acids and achiral glycine. The porosity of DTD’s active site to glycine in turn leads to active misediting of Gly-tRNA^Gly^, thereby presenting a scenario in which the Gly-tRNA^Gly^ pool in the cell is depleted. We further demonstrate that this apparent glycine “misediting paradox” created by DTD is effectively resolved through protection of Gly-tRNA^Gly^ by elongation factor thermo unstable (EF-Tu). However, our biochemical and cell-based assays reveal that DTD concentration in the cell is at a tipping point, and any increase in DTD levels makes it toxic for the cell. Therefore, the study clearly underlines a strict requirement for a tight control of DTD’s expression and brings to the fore novel perspectives in terms of its regulation in all life forms.

## Results

### NMR Spectroscopy–Based Binding Studies Indicate Glycine Binding

**Fig 1:**
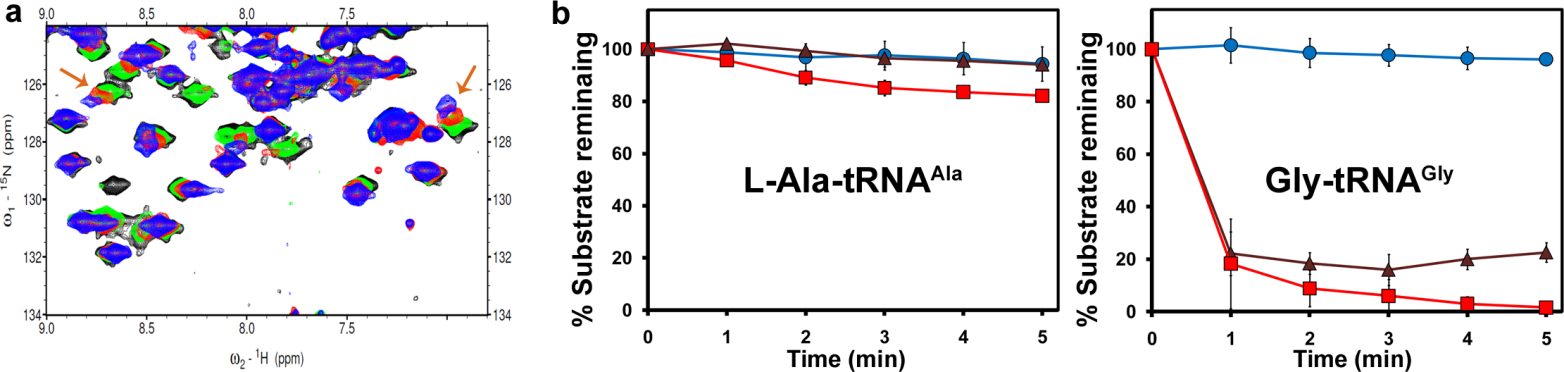
DTD binds and deacylates achiral glycine. **(a)** Excerpts of overlay of 2D ^15^N-^1^H Transverse Relaxation Optimized Spectroscopy obtained with 0.2 mM PfDTD (black) when titrated with 3 mM each of L-Ala3AA (green), Gly3AA (red), and D-Ala3AA (blue). Arrows indicate chemical shift perturbations. **(b)** Deacylation of L-Ala-tRNA^Ala^ and Gly-tRNA^Gly^ by buffer (blue circle), 50 nM EcDTD (brown triangle), and 500 pM PfDTD (red square). Error bars indicate one standard deviation from the mean. The underlying data of panel **(b)** can be found in S1 Data.

To test the mechanistic model of L-chiral rejection even for the smallest chiral proteinogenic amino acid, alanine, nuclear magnetic resonance (NMR) spectroscopy–based 2D ^15^N-^1^H Transverse Relaxation Optimized Spectroscopy experiments were performed with ^15^N-PfDTD (DTD from *Plasmodium falciparum*) (UniProt ID: Q8IIS0). Post-transfer substrate analogs, D- and L-alanyl-3′-aminoadenosine (D- and L-Ala3AA), were used, in which the aminoacyl moiety is covalently linked to the 3′-carbon of the adenosine moiety through an amide linkage instead of ester linkage, thus mimicking adenosine 76 (A76) at the 3′-terminal of aminoacyl-tRNA. While the data indicated binding of D-Ala3AA but not of L-Ala3AA, surprisingly, titration with glycyl-3′-aminoadenosine (Gly3AA) showed noticeable chemical shift perturbations, suggesting binding of the achiral substrate (Figs 1A and S1A–S1F). To further gain a better understanding, we screened two other D-amino acid analogs, D-aspartyl-3′-aminoadenosine and D-seryl-3′-aminoadenosine, and both did not show any binding (S1G and S1H Fig). The above indications were intriguing, considering the fact that it has been noted earlier that deacylation by EcDTD (DTD from *Escherichia coli*) (UniProt ID: P0A6M4) is 2-fold more efficient on D-Asp-tRNA^Asp^ (k_cat_/K_m_ = 12 μM^-1^ s^-1^) than on D-Tyr-tRNA^Tyr^ (k_cat_/K_m_ = 6 μM^-1^ s^-1^) [14]. It should be mentioned here that binding of an analog, or the lack of it, cannot be construed as an indication of biochemical activity against the corresponding substrate, which is the aminoacyl-tRNA. However, the above binding studies prompted us to probe further into the mechanism of chirality-based selection/rejection by DTD.

### DTD Can Efficiently Deacylate Gly-tRNA^Gly^

We generated Gly-tRNA^Gly^ substrate to test directly both EcDTD and PfDTD for their activity against it. Strikingly, deacylation assays showed that DTD can act efficiently on Gly-tRNA^Gly^ (Fig 1B). We studied the kinetics of EcDTD on Gly-tRNA^Gly^ and found that the k_cat_ and K_m_ values of the enzyme were 10 s^-1^ and 1 μM, respectively. The kinetic constants of EcDTD for Gly-tRNA^Gly^ are comparable to those reported for other substrates, namely D-Tyr-tRNA^Tyr^, D-Trp-tRNA^Trp^, and D-Asp-tRNA^Asp^ (S1 Table). This brings forth a novel enzyme design paradigm, clearly indicating that there is no selection of D-amino acid, as the chiral proofreading site does not discriminate between achiral and D-chiral substrates. To further test whether L-alanine will be accommodated in the binding pocket, we generated L-Ala-tRNA^Ala^ substrate. While Gly-tRNA^Gly^ is deacylated readily by EcDTD and PfDTD at a concentration of 50 nM and 500 pM, respectively, the assays did not result in any noticeable deacylation of L-Ala-tRNA^Ala^ (Fig 1B). Notably, a 100-fold increase in DTD concentration resulted in marginal deacylation of L-Ala-tRNA^Ala^, while for significant deacylation of L-Ala-tRNA^Ala^, a 10,000-fold increase in the concentration of PfDTD was required (S2 Fig). The above results clearly suggest that while the chiral proofreading site is impermeable to L-amino acids with larger side chains, L-alanine can squeeze in, albeit with a significantly lower efficiency, as explained later. Therefore, DTD can discriminate effectively against even the smallest L-amino acid, despite the fact that the chiral proofreading site is completely porous to achiral glycine.

### Gly-tRNA^Gly^ Deacylation by DTD Is a Global Phenomenon

**Fig 2:**
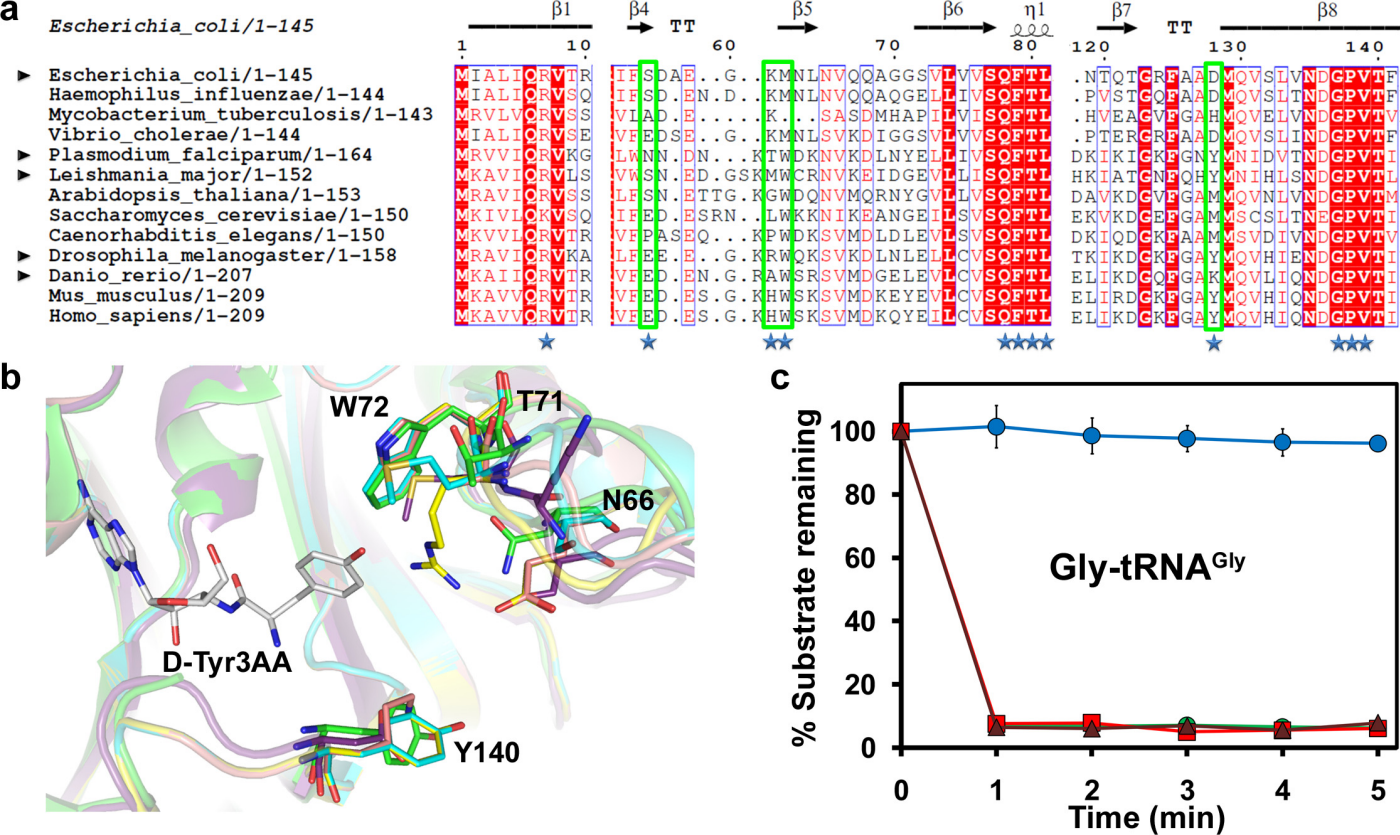
Glycine misediting by DTD is a universal phenomenon. **(a)** Structure-based multiple sequence alignment of DTD from various organisms highlighting the variant residues in the active site. Residues marked with stars are within 6 Å radius of D-Tyr moiety of D-Tyr3AA. Amino acids enclosed in green boxes are varying in DTDs across different organisms. DTDs from organisms indicated by black arrowheads have been tested for biochemical activity in the current study. **(b)** Non-conserved residues in the chiral proofreading site of various DTDs—*Plasmodium falciparum* (green; PDB id: 4NBI), *Escherichia coli* (violet; PDB id: 1JKE), *Leishmania major* (cyan; model), *Drosophila melanogaster* (yellow; model), and *Danio rerio* (pink; model). **(c)** Deacylation of Gly-tRNA^Gly^ by buffer (blue circle), 50 nM LmDTD (green circle), 50 nM DmDTD (red square), and 50 nM DrDTD (brown triangle). Error bars indicate one standard deviation from the mean. The underlying data of panel **(c)** can be found in S1 Data.

To ascertain the universality of Gly-tRNA^Gly^ deacylation by DTD, we analysed the active site of DTDs from diverse organisms. Structure-based multiple sequence alignment and homology modelling of DTD from both bacterial and eukaryotic species revealed that in a radius of approximately 6 Å from the side chain of D-tyrosyl-3′-aminoadenosine (D-Tyr3AA) in the amino acid–binding pocket, four out of 12 residues were not conserved, the variations being more in the eukaryotic proteins (Fig 2A and 2B). Such differences may not only affect the binding of a substrate to the enzyme’s active site, but also the enzyme’s biochemical activity. Thus, we decided to probe DTDs from different eukaryotes—one unicellular, one invertebrate, and one vertebrate—for the universality of glycine deacylation. We therefore overexpressed, purified, and tested DTDs from *Leishmania major* (LmDTD) (UniProt ID: Q4Q1E7), *Drosophila melanogaster* (DmDTD) (UniProt ID: Q9VGP0), and *Danio rerio* (DrDTD) (UniProt ID: Q6DH41), in addition to DTD from *P*. *falciparum*. Biochemical assays clearly showed that the variations in the amino acid–binding pocket do not affect DTD’s activity on Gly-tRNA^Gly^, as all of them readily deacylated Gly-tRNA^Gly^, suggesting that it is a global phenomenon (Fig 2C).

### Glycine Prefers the Flipped Orientation in the Active Site Pocket

**Fig 3:**
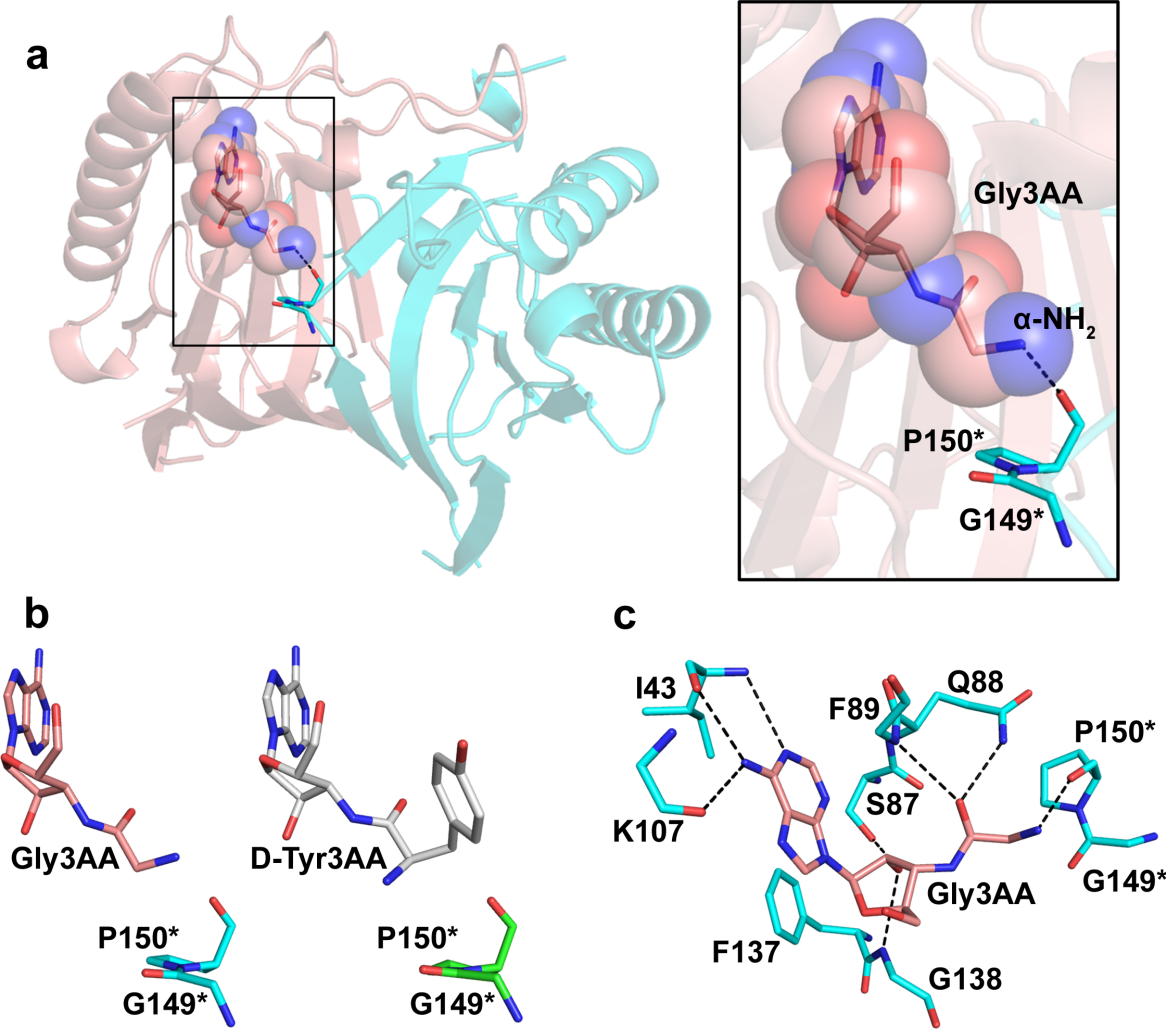
Glycine binding mode in the chiral proofreading site of DTD. **(a)** Co-crystal structure of PfDTD with Gly3AA showing the capture of the ligand. **(b)** Comparison of PfDTD+Gly3AA complex with PfDTD+D-Tyr3AA complex (PDB id: 4NBI) showing the flipped orientation of the α-NH_2_ group of Gly3AA. **(c)** Network of interactions of Gly3AA with active site residues of PfDTD. Residues indicated by * are from the dimeric counterpart.

**Fig 4:**
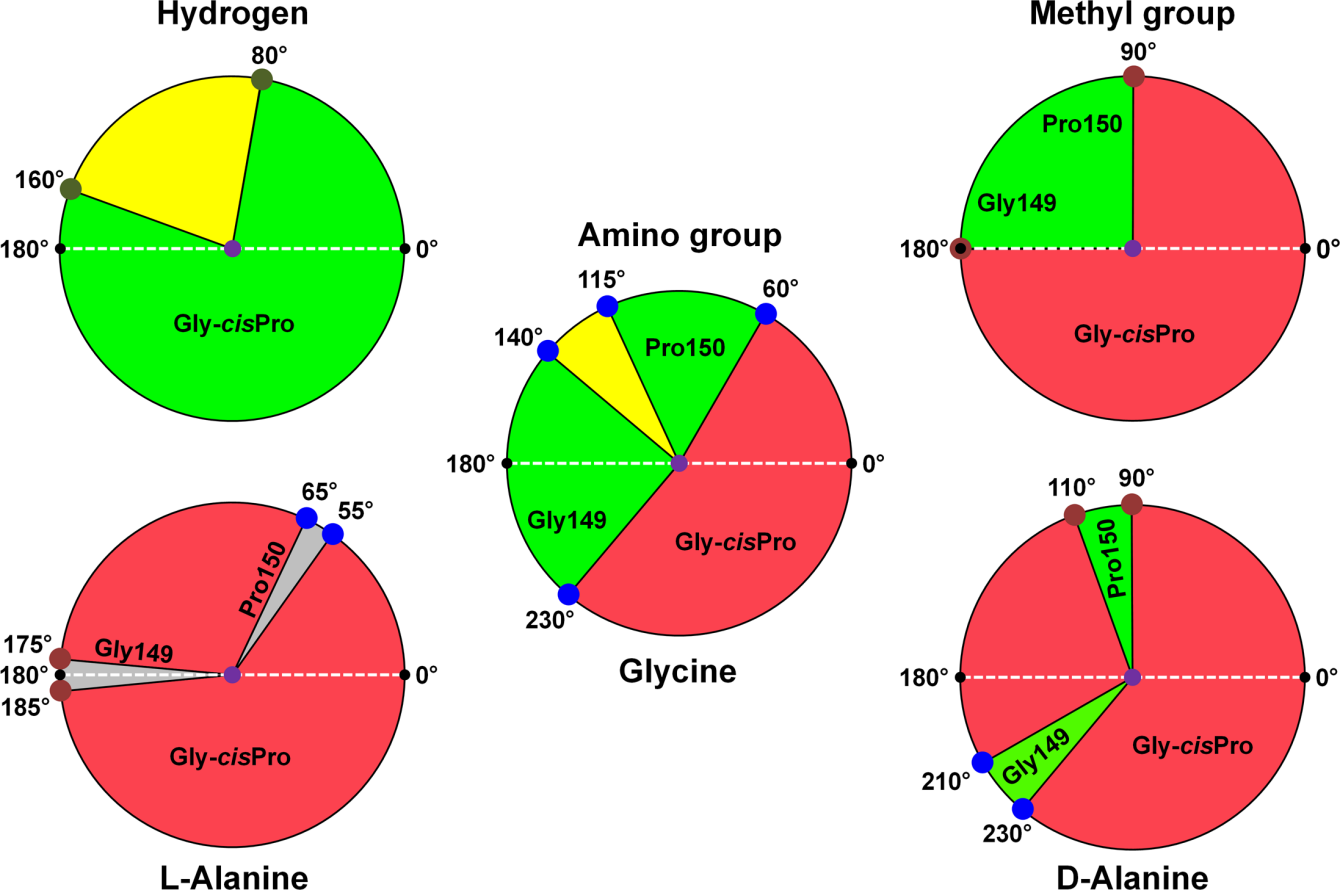
Mechanistic design principle of the active site of DTD. Schematic showing the relative orientations of different groups attached to Cɑ of the ligand when viewed down Cα–C bond (centre of the wheel). The carboxyl plane of the ligand, represented as a white dashed line, with the two oxygen atoms at 0° and 180°, has been taken as the reference for calculating the angular sweeps. The top two wheels and the middle wheel represent each group attached to the chiral centre individually, while the bottom two wheels and the middle wheel represent each of the three ligands—L-alanine (with methyl and amino groups), glycine (with amino group only), and D-alanine (with methyl and amino groups). The following colour coding for different groups has been used: H: dark green, NH_2_: blue, CH_3_: brown. The delineation of regions is based on the following distance criteria from Gly149 O or Pro149 O for each group: H, 2.0 Å to 3.0 Å; NH_2_, 2.5 Å to 3.5 Å; CH_3_, 3.0 Å to 4.0 Å [34]. Regions falling within the range are favoured (green: region of interaction), above the range are allowed (yellow: region of no interaction), and below the range are disallowed (red: region of steric clash). In the case of L-alanine, the amino acid can be squeezed in the pocket with the methyl group positioned at 3.0 Å from the Gly149 O at 180° and the amino group at 2.5 Å from Pro150 O at 60°; hence, the possible allowed region for L-alanine has been shaded grey, and any other positioning of the methyl group would result in more serious clashes. Calculations are based on protein-based superimposition of all the monomers of PfDTD+Gly3AA complex and the average Cɑ, C, and O positions of the glycyl moiety. For distance measurements, all the observed orientations of Gly-*cis*Pro motif are taken into consideration, thereby accounting for the plasticity of the active site.

Since Gly-tRNA^Gly^ was found to be efficiently deacylated by multiple DTDs from different organisms, it became imperative to further dissect the mechanism by which DTD achieves its enantioselectivity. We solved the co-crystal structure of PfDTD with Gly3AA at 2.1 Å resolution in *P*2_1_ space group (Table 1). The crystal form had eight monomers in the asymmetric unit and, therefore, allowed us to compare eight independent observations of glycine captured by DTD’s proofreading site. It was intriguing to find that the electron density for the glycyl moiety was weaker when compared to that for adenine and ribose, indicating an inherent flexibility of glycine when it sits in the active site pocket (S3 Fig). This has also been indicated by the comparison of atomic B-factors of Gly3AA analog in all the monomers (S4 Fig). However, it is important to note that the carbonyl oxygen of glycine is rigidly fixed by a DTD fold-specific interaction with main chain N of Phe89 and by its interaction with Nε of Gln88 (Fig 3C and S2 Table). Thus, the flexibility in glycine was observed only for its α-NH_2_ group. Analysis of the electron density maps revealed that the α-NH_2_ group has a preference for the flipped orientation (i.e., the position in which Cβ was seen in D-Tyr3AA complexes), despite its inherent flexibility (Figs 3A, 3B and S3). Consequently, we placed the α-NH_2_ group in the flipped orientation in all eight monomers before the final round of refinement. Therefore, the fact that the α-NH_2_ group was seen oriented toward the carbonyl O of Gly149 in D-Tyr3AA complexes was not due to any selection of the amino group, but it was the only way it could be accommodated when compared to the constraints of placing a methyl group in that position, as discussed later. We have performed a thorough atom-by-atom analysis of ligands in the chiral proofreading site and proposed a more elaborate and in-depth model for DTD’s mechanism of enantioselectivity (Fig 4). As discussed later, the DTD’s active site is designed for binding anything but L-enantiomeric amino acids attached to tRNA.

**Table 1:** Crystallographic data collection and refinement statistics.

	PfDTD+Gly3AA
**Data collection**	
Space group	*P*2_1_
Cell dimensions	
*a*, *b*, *c* (Å)	57.16, 86.44, 138.16
*ɑ*, *β*, *γ* (°)	90.00, 93.39, 90.00
Resolution (Å)	25.00–2.10 (2.18–2.10)*
*R*_sym_ or *R*_merge_ (%)	11.2 (87.6)
*I/σI*	14.7 (2.5)
Completeness (%)	96.5 (99.5)
Redundancy	4.9 (5.0)
**Refinement**	
Resolution (Å)	25.00–2.10 (2.18–2.10)
No. of reflections	71448
*R*_work_ / *R*_free_ (%)	22.01/26.96
No. of atoms	10407
Protein	10044
Ligand/ion	184
Water	179
*B*-factors (Å^2^)	
Protein	59.3
Ligand/ion	70.6
Water	58.8
R.m.s. deviations	
Bond lengths (Å)	0.007
Bond angles (°)	1.194

One crystal was used for data collection and structure solution.

*Values in parentheses are for highest-resolution shell.

To test whether the mode of ligand binding in the chiral proofreading site was biologically relevant and was not a crystallographic artifact, two mutants of the adenine-binding site were generated and tested for biochemical activity. In both EcDTD and PfDTD, an invariant phenylalanine (EcDTD Phe125 and PfDTD Phe137), which holds the adenine ring through stacking interaction (Figs 3C and S5A), was mutated to alanine. In the second mutant, an alanine (EcDTD Ala102 and PfDTD Ala112) was replaced by a bulkier phenylalanine to sterically abrogate the binding of adenine in the pocket (S5A Fig). Both phenylalanine-to-alanine and alanine-to-phenylalanine mutants of EcDTD as well as PfDTD were found to be biochemically inactive on Gly-tRNA^Gly^ (S5B Fig), thereby validating that the mode of ligand binding as observed in the crystal structure represents the true binding of the actual, full-size substrate (i.e., Gly-tRNA^Gly^). In our previous study, we had shown that these mutants were also inactive on D-Tyr-tRNA^Tyr^, thus authenticating the binding mode of the analog D-Tyr3AA in the chiral proofreading site [15].

### EF-Tu Confers Protection on Gly-tRNA^Gly^ against DTD

**Fig 5:**
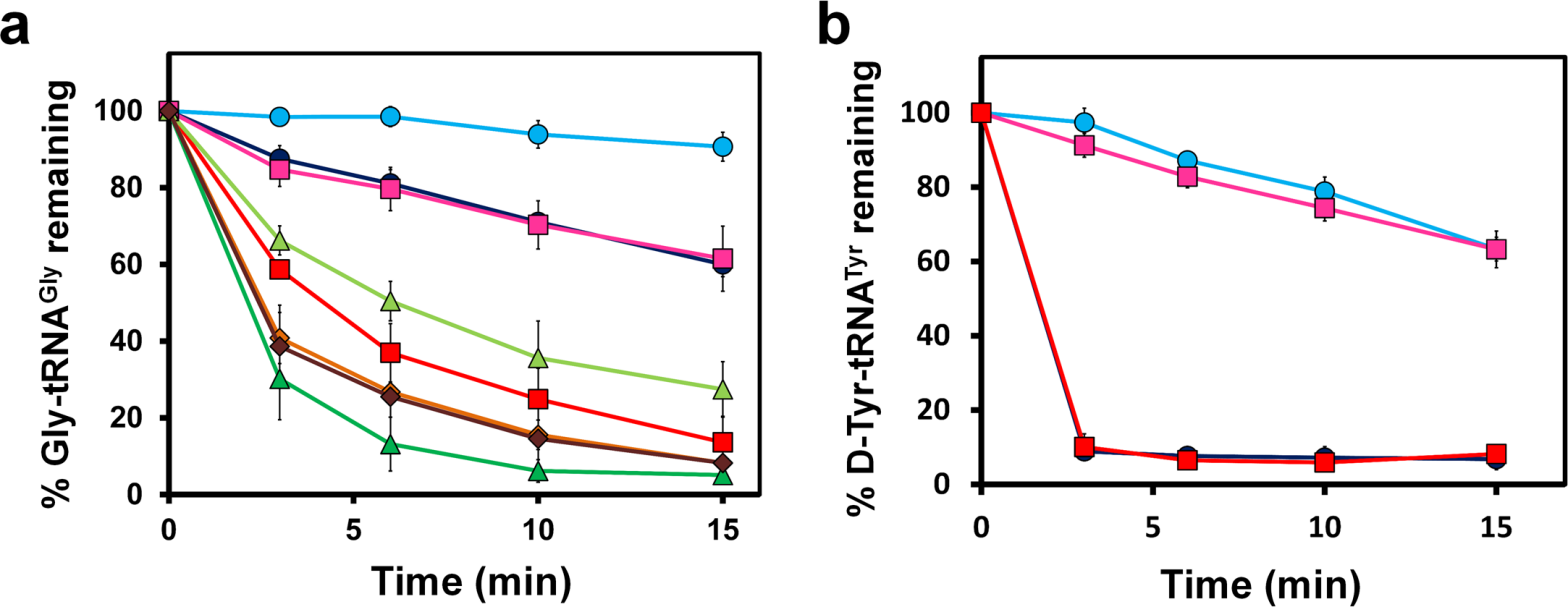
EF-Tu confers protection on Gly-tRNA^Gly^. **(a)** Deacylation of Gly-tRNA^Gly^ in the presence of unactivated EF-Tu (dark blue circle), activated EF-Tu (light blue circle), unactivated EF-Tu and 5 nM EcDTD (red square), activated EF-Tu and 5 nM EcDTD (pink square), unactivated EF-Tu and 10 nM EcDTD (dark green triangle), activated EF-Tu & 10 nM EcDTD (light green triangle), unactivated EF-Tu and 20 nM EcDTD (dark brown diamond), and activated EF-Tu and 20 nM EcDTD (light brown diamond). **(b)** Deacylation of D-Tyr-tRNA^Tyr^ in the presence of unactivated EF-Tu (light blue circle), activated EF-Tu (pink square), unactivated EF-Tu and 5 nM EcDTD (dark blue circle), activated EF-Tu and 5 nM EcDTD (red square). Error bars indicate one standard deviation from the mean. The underlying data can be found in S1 Data.

DTD’s gratuitous activity on glycine is deleterious to the cell, as it will deplete the Gly-tRNA^Gly^ pool, thereby causing translation to slow down or even stop altogether. We hypothesised that EF-Tu (UniProt ID: Q5SHN6 for *tufA* gene product in *Thermus thermophilus*) should be able to confer protection on Gly-tRNA^Gly^ against DTD, since EF-Tu acts as a delivery protein to transfer aminoacyl-tRNAs to the ribosome. Therefore, we performed competition (deacylation) assays with DTD and EF-Tu, and found that 2 μM of EF-Tu offers adequate protection to glycine from DTD when the latter is present at 5 nM concentration (Fig 5A). A control reaction done with D-Tyr-tRNA^Tyr^ showed that such a protection is not conferred by EF-Tu on the D-substrate (Fig 5B). Considering only 10%–15% of the activated form of EF-Tu (i.e., GTP-bound EF-Tu) that is present in the entire EF-Tu pool [35,36] in the experimental conditions tested, the effective EF-Tu concentration that provided protection was 200–300 nM. It is important to note that EF-Tu is abundantly present in the *E*. *coli* cell, whereas DTD is a low-expressing protein [37,38]. Moreover, an inspection of several expression databases (dictyExpress, Saccharomyces Genome Database, FlyBase) clearly suggests that DTD is kept at low levels universally, whereas EF-Tu (or elongation factor 1 alpha, the eukaryotic homolog of EF-Tu) is highly expressed. The number of EF-Tu and DTD molecules per *E*. *coli* cell is about 100,000 and 500, respectively [38], i.e., a 200-fold difference. However, since there are 20 different aminoacyl-tRNAs with which EF-Tu binds, there will be effectively about 5,000 EF-Tu molecules available for Gly-tRNA^Gly^. This implies that the difference between EF-Tu and DTD for Gly-tRNA^Gly^ protection is approximately 10-fold. In our deacylation assays, EF-Tu conferred protection only when DTD was present at 5 nM (40- to 60-fold difference), but the protection was completely relieved when DTD’s concentration was increased to 20 nM (10- to 15-fold difference). At 10 nM DTD (20- to 30-fold difference), protection was only partial (Fig 5A). The observed discrepancy between cellular numbers and biochemical assays is probably because in the cell, molecular crowding and local concentrations may have effect in providing protection to Gly-tRNA^Gly^. Nevertheless, what these data clearly indicate is that the concentration of DTD in the cell is at a tipping point and must be tightly regulated, and why DTD is kept at low levels in the cell.

### DTD Overexpression Causes Cellular Toxicity

**Fig 6:**
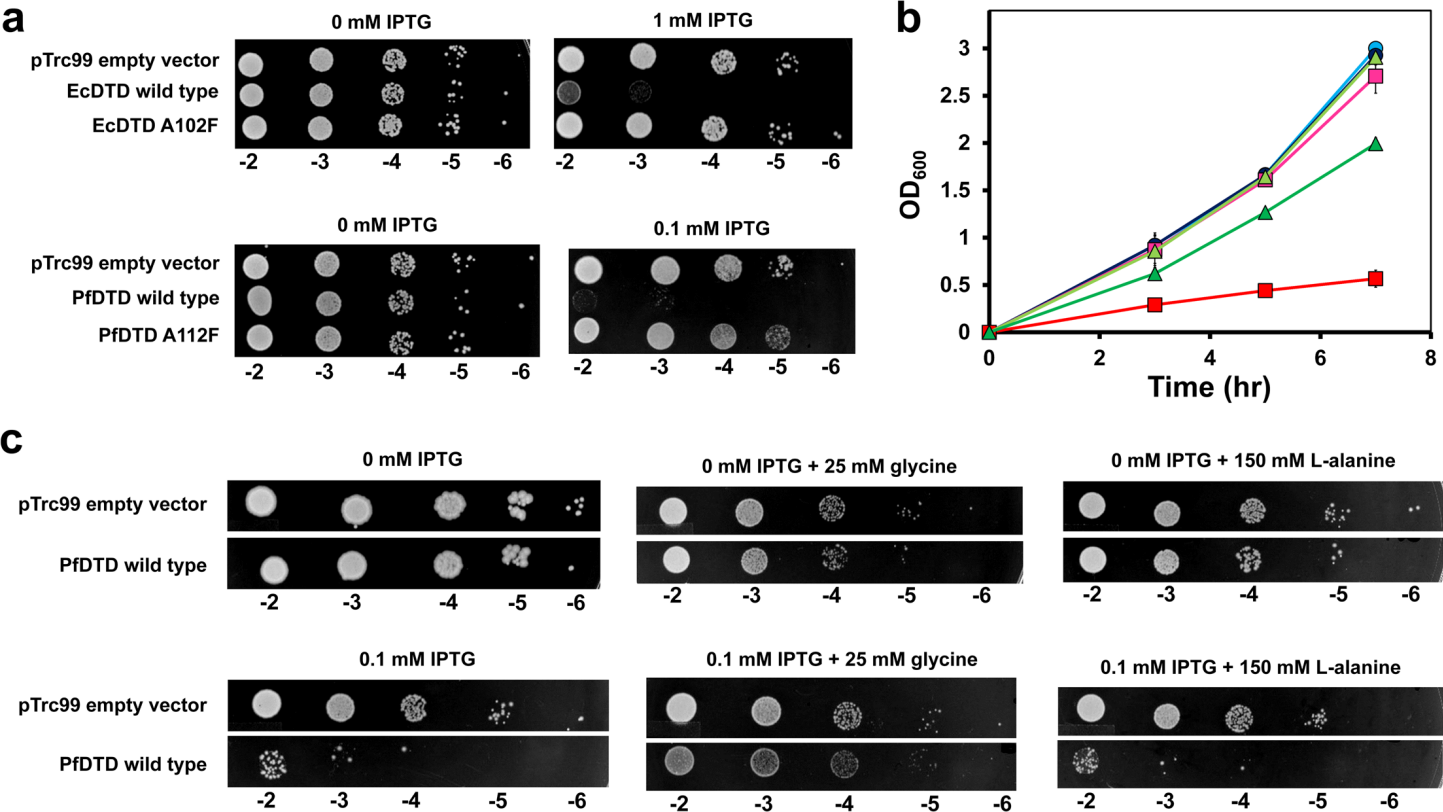
DTD overexpression causes cellular toxicity. **(a)** Spot dilution assay of *E*. *coli* K12Δ*dtd*::Kan complemented with pTrc99 empty vector, EcDTD/PfDTD wild-type, or EcDTD A102F/PfDTD A112F. **(b)** Growth curve of *E*. *coli* K12Δ*dtd*::Kan complemented with pTrc99 empty vector uninduced (light blue circle) or induced (dark blue circle), PfDTD wild-type uninduced (pink square) or induced (red square), and PfDTD A112F uninduced (light green triangle) or induced (dark green triangle); induction with 0.1 mM IPTG. Error bars indicate one standard deviation from the mean. (**c**) Spot dilution assay of *E*. *coli* K12Δ*dtd*::Kan complemented with pTrc99 empty vector or PfDTD wild-type. The top panel represents uninduced cultures (i.e., 0 mM IPTG), whereas the bottom panel corresponds to cultures induced with 0.1 mM IPTG. The cultures are unsupplemented or supplemented with 25 mM glycine or with 150 mM L-alanine. The underlying data of panel **(b)** can be found in S1 Data.

DTD’s activity on Gly-tRNA^Gly^ meant that DTD overexpression in the cell should lead to Gly-tRNA^Gly^ depletion and, hence, cellular toxicity. We therefore performed in vivo assays to test this hypothesis. We used a *dtd*-null strain of *E*. *coli* (K12Δ*dtd*::Kan) and complemented it with *dtd* from *E*. *coli* (NCBI Gene ID: 948378) or from *P*. *falciparum* (NCBI Gene ID: 810646). Spot dilution assays with both systems clearly showed that DTD overexpression induces significant cellular toxicity (Fig 6A). To rule out toxicity due to protein overexpression, control experiments were performed with the biochemically inactive mutant of DTD, EcDTD A102F and PfDTD A112F, in which the bulkier residue protrudes into the adenine-binding site meant for the 3′-terminal adenosine of tRNA to which the amino acid is attached (S5A Fig) [15]. Assays with both mutant proteins showed no toxic effect of the same on cell growth (Fig 6A), suggesting clearly that the toxicity observed is a direct result of DTD's enzymatic function rather than any off-target interaction in the cellular scenario. Even growth curves of PfDTD showed marked toxicity of the wild-type, whereas the inactive mutant exhibited only marginal retardation in growth (Fig 6B).

We further tested whether glycine supplementation will rescue the cellular toxicity by possibly promoting aminoacylation of the free tRNA^Gly^. Supplementation assays performed with 25 mM glycine showed significant rescue from cellular toxicity by PfDTD (Fig 6C). Control experiments carried out with 150 mM L-alanine failed to rescue the cells from PfDTD’s toxicity (Fig 6C). To rule out strain-specific effects, toxicity assays were also performed with *E*. *coli* BL21(DE3), which gave similar results (S6 Fig). Thus, our in vivo studies further suggested that higher DTD levels can lead to cellular toxicity by depleting the Gly-tRNA^Gly^ pool, and that under normal growth conditions, EF-Tu confers protection on the achiral substrate against DTD.

### DTD Is an RNA-Based Catalyst

**Fig 7:**
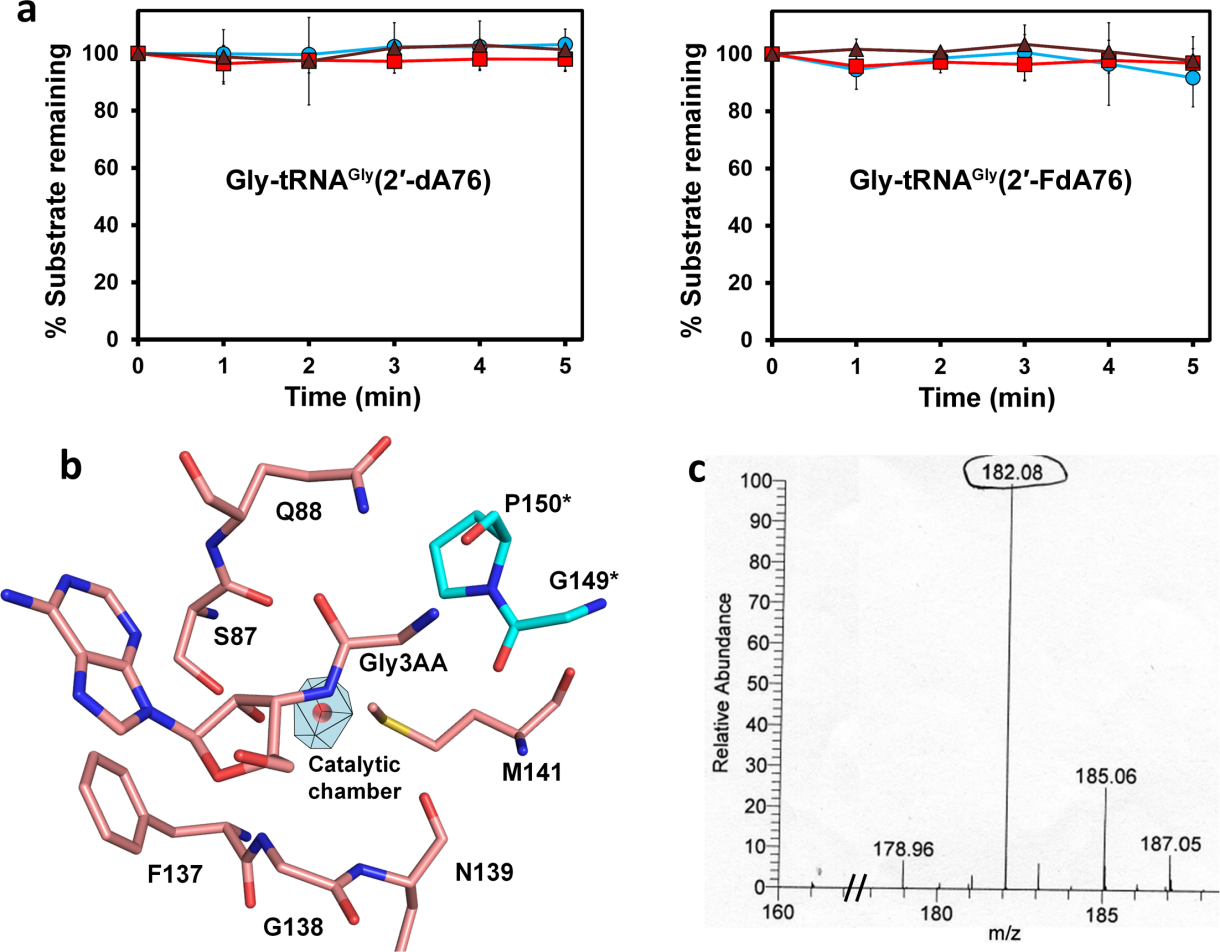
DTD is an RNA-based catalyst. **(a)** Deacylation of Gly-tRNA^Gly^(2'-dA76) and Gly-tRNA^Gly^(2'-FdA76) by buffer (blue circle), 5 μM EcDTD (brown triangle), and 5 μM PfDTD (red square). Error bars indicate one standard deviation from the mean. **(b)** Schematic showing the space (catalytic chamber) in the active site of PfDTD, which can accommodate a water molecule (catalytic water, depicted as red sphere), although the water molecule is not observed in the crystal structure. Residues indicated by * are from the dimeric counterpart. **(c)** Mass spectrometry analysis of the deacylation product (amino acid) after carrying out deacylation of D-Tyr-tRNA^Tyr^ with PfDTD in H_2_O^18^. The underlying data of panel **(a)** can be found in S1 Data.

We also showed earlier that the active site residues of DTD, potentially capable of performing catalysis, are dispensable [15]. Furthermore, our previous studies on the structural homolog of DTD, N-terminal editing domain (NTD) of archaeal ThrRS, had suggested a catalytic role of 2′-OH of terminal adenosine (A76) [39,40]. Earlier, we were unable to test the above hypothesis, as tyrosyl-tRNA synthetase charges L-/D-tyrosine on tRNA^Tyr^ at 2′-OH, whereas ThrRS requires 2′-OH to charge L-serine/L-threonine on tRNA^Thr^ at 3′-OH. We could generate 3'-end modifications of tRNA^Gly^ with A76 being replaced by either 2′-deoxyadenosine (2′-dA76) or 2′-fluoro-2′-deoxyadenosine (2′-FdA76) and could also charge the modified tRNAs with glycine using glycyl-tRNA synthetase. Employing this strategy to test our hypothesis on NTDs, we have very recently been successful in proving that NTDs can catalyse deacylation of their substrates only with the assistance of 2′-OH of tRNA [41]. We used the same strategy to substantiate our hypothesis on DTD. When tested biochemically, even a 100-fold and a 10,000-fold excess of EcDTD and PfDTD, respectively, failed to deacylate 3′-end modified substrates, thereby validating the hypothesis (Fig 7A).

On the basis of structural analyses of NTD, we proposed that a catalytic water molecule (observed in the crystal structures of NTD), positioned between the 2′-OH of A76 and the carbonyl carbon of the ligand, would be activated by 2′-OH to mount a nucleophilic attack and cleave the scissile ester bond [39,40]. Surprisingly, the catalytic water molecule was never observed in any of the ligand-bound crystal structures of DTD, i.e., neither in D-Tyr3AA complexes from our earlier work [15] nor in the Gly3AA complex presented here. Nevertheless, the space in the DTD pocket is always above the threshold volume of approximately 21 Å^3^ required for accommodating a water molecule (Fig 7B and S3 Table). We performed deacylation of D-Tyr-tRNA^Tyr^ by PfDTD in H_2_O^16^ and H_2_O^18^, and the released product (amino acid) was subjected to mass spectrometric analysis. The deacylation product of the experiment carried out in H_2_O^16^ gave a peak corresponding to normal D-tyrosine, whereas the product of the reaction performed in H_2_O^18^ gave a peak having an increase in mass by 2 Da (Figs 7C and S7). Thus, we now provide the direct proof of DTD acting as an RNA-based catalyst wherein the 2′-OH of the ribose activates a water molecule for the deacylation reaction. Therefore, the DTD-like fold is employed by nature in two distinct functional contexts and complementarily in the three domains of life: (1) as a D-amino acid removal system from tRNA in bacteria and eukaryotes, and (2) as the editing domain of ThrRS from archaea, using RNA for catalysis in both cases. Taken together, with our work on the archaeal editing domain of ThrRS [39–42], the above data suggest a primordial origin for the fold that acts at the RNA–protein interface rather than using side chains for specificity and catalysis.

## Discussion

In this work, we have provided a unique dimension to our understanding of DTD’s mechanism of enantioselection and its proofreading activity. Unlike earlier, when we had proposed an active D-chiral selection by the Gly-*cis*Pro motif of DTD, we have now provided evidence that strict L-chiral rejection, rather than D-chiral selection, is the one and only fundamental design principle that DTD uses to achieve remarkable configurational specificity. The mode of capture of carbonyl oxygen, which is a conserved feature of DTD-like fold, ensures that only three modes are possible for the groups attached to the chiral carbon [15]. Interestingly, the Gly-*cis*Pro motif that was thought to select the D-enantiomer provides the underlying structural basis for L-enantiomer rejection. Our analysis of placement of atoms on the basis of geometric considerations with respect to the chiral centre (Cɑ), both in isolation and with other atoms, shows that, for L-amino acids, the methyl group of L-alanine can be squeezed in one orientation only, thereby resulting in significant reduction in catalytic efficiency (Fig 4). The situation with L-alanine is typically a case of forcing an atom in an unfavourable environment, and the significant cost for the same is beautifully exemplified by the marginal activity on L-Ala-tRNA^Ala^ only upon increasing the concentration of DTD by 100-fold (S2 Fig).

**Fig 8:**
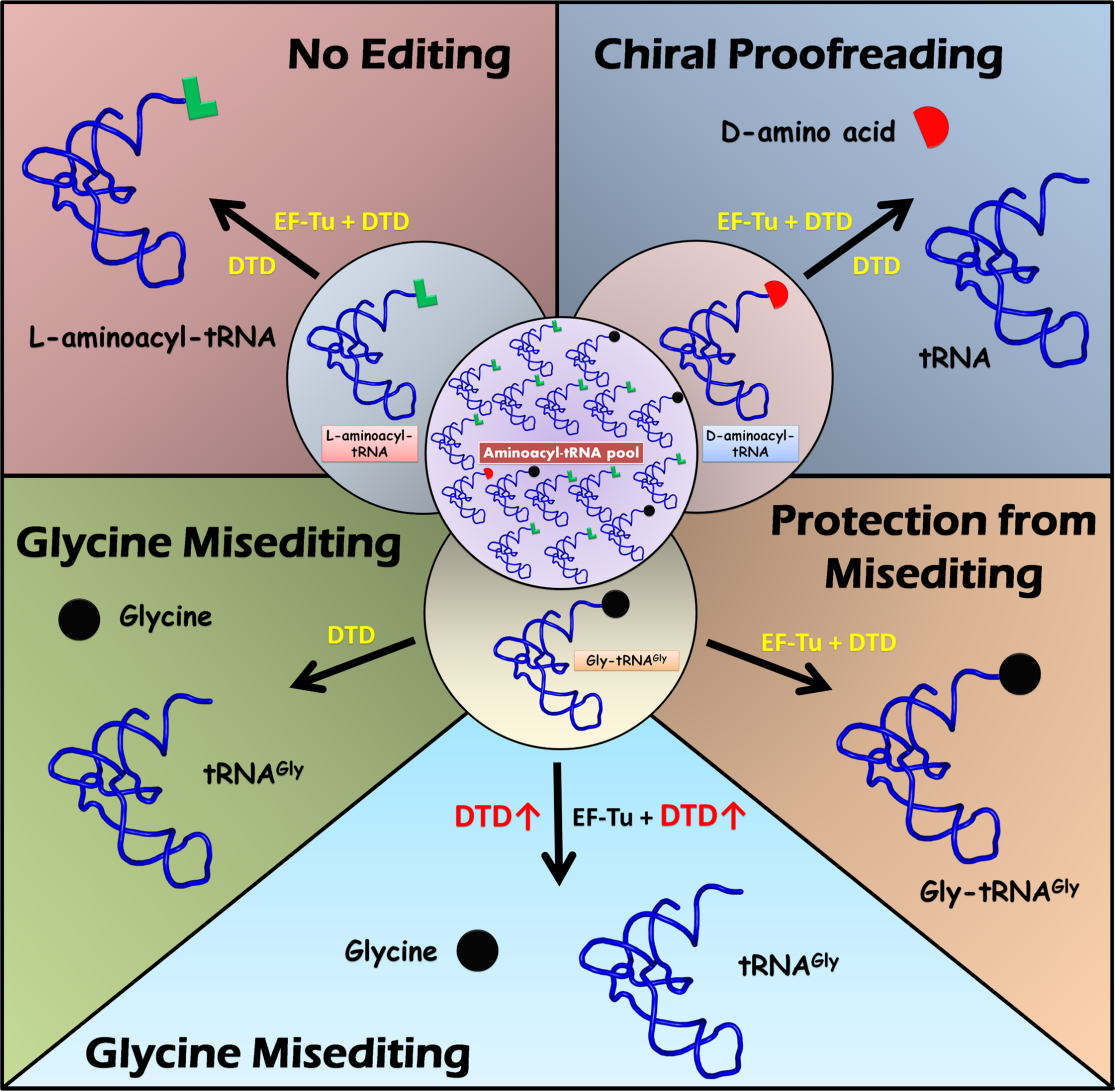
Model for protection of Gly-tRNA^Gly^ by EF-Tu. In the cell, the aminoacyl-tRNA pool comprises mostly L-aminoacyl-tRNAs, some Gly-tRNA^Gly^, and very few D-aminoacyl-tRNAs (central circle). L-aminoacyl-tRNAs are not acted upon by DTD and, hence, no editing (top left). DTD efficiently decouples D-amino acids mischarged on tRNAs (chiral proofreading), even in the presence of abundant EF-Tu (top right). Gly-tRNA^Gly^ can be edited by DTD in the absence of EF-Tu (misediting; bottom left), which is, however, effectively prevented by EF-Tu (protection from misediting; bottom right). Under conditions in which DTD levels are relatively high, i.e., DTD is overexpressed, protection is relieved, leading to glycine misediting and cellular toxicity (bottom middle).

On the other hand, if there were any selection of the amino group of D-amino acids, as observed in the case of D-Tyr3AA complexes [15], the amino group of glycine moiety in Gly3AA complex would be expected to occupy the same position. In contrast, it prefers majorly the position occupied by the Cβ in D-Tyr3AA complexes, clearly showing a lack of strong selection of the amino group in the opposite orientation (Fig 3B). Hence, the lack of rejection in the binding pocket leads to a significant deacylation of Gly-tRNA^Gly^. The glycine “misediting paradox” is thus generated by the mechanistic design of a single enzyme that rejects every L-amino acid and does not positively select any D-amino acid, irrespective of the side chain (Fig 8). The D-amino acid binding mode, therefore, is a happenstance, as DTD’s proofreading site design is unable to discriminate between glycine and D-amino acids. More importantly, glycine misediting by DTD is not a case of a smaller substrate binding in a larger pocket, which is seen routinely in any protein–ligand interaction system, but due to a lack of any selection of any of the D-chiral substrates. The design of a single deacylase to deacylate any one of the 19 D-amino acids attached to any tRNA is the inherent reason behind this misediting phenomenon. The situation is unlike in the case of different D-amino acid oxidases which are specific for different chemical classes of amino acids, like hydrophobic or negatively charged [43]. Moreover, D-amino acid oxidases do not act on glycine, and a separate and distinct oxidase is recruited to act on achiral glycine [44,45].

Physiologically, the high activity of DTD on Gly-tRNA^Gly^ is unwarranted, leading to the latter’s misediting and, hence, could probably retard or even completely inhibit protein synthesis. This may eventually cause growth defects and cellular toxicity. DTD’s cross-reactivity with glycine, therefore, poses a clear and present threat to the cell, which recruits EF-Tu to preclude the deleterious effect of DTD on the translational apparatus. In fact, EF-Tu is one of the most highly expressed proteins in the cell, whereas DTD levels are usually kept low [37,38]. However, higher concentrations of DTD can overcome protection conferred on Gly-tRNA^Gly^ by EF-Tu (Fig 5A), and this can have serious biological ramifications. Here, we provide direct experimental evidence that DTD has significant activity on the achiral susbtrate, which provides a rationale for the observed toxicity in the cell-based assays (Figs 1, 3 and 6).

A concomitant increase in EF-Tu levels in the cell to neutralise the toxic effect of DTD when the latter is overexpressed is an obvious strategy one can resort to. However, EF-Tu overexpression is technically challenging and difficult to achieve for three fundamental reasons: (a) EF-Tu is house-keeping and abundantly expressing (approximately 5%–10% of total proteins in *E*. *coli*) [46]; (b) apart from translation, EF-Tu is required for several other important physiological processes such as molecular chaperoning [47,48], maintenance of cytoskeleton and cell shape [49], and protein disulphide isomerisation [50]; and (c) EF-Tu is an autogenous regulator that regulates its own expression levels [51,52]. Considering the multifaceted nature of EF-Tu, perturbing cellular EF-Tu levels drastically is expected to unfavourably affect multiple cellular networks and pathways and, eventually, cell physiology and homeostasis. Accordingly, it has been shown that raising EF-Tu levels in the cell through overexpression by approximately 75% negatively affects cell growth [52]. Likewise, decreasing EF-Tu in the cell by even less than half of the normal levels via knocking out one of the two *tuf* genes (*tufA* and *tufB*) results in similar effects [52,53,54]. The fact that EF-Tu concentration in the cell cannot be changed significantly over its normal levels naturally necessitates tight regulation of DTD in the cell.

Furthermore, rescue from toxicity through glycine supplementation corroborates the notion that DTD’s toxicity stems from its activity on Gly-tRNA^Gly^. Thus, regulation of DTD’s expression becomes imperative for two obvious reasons: (1) Gly-tRNA^Gly^ expectedly is much more in number than D-aminoacyl-tRNAs, and (2) glycine is one of the most abundant amino acids found in proteins; any reduction in Gly-tRNA^Gly^ levels will adversely impact translation rate. Intriguingly, DTD expression has been shown to be relatively high in certain tissues, such as neurons, in which some D-amino acids are present in higher amounts [55]; for instance, D-serine and D-aspartate, which act as neurotransmitters, are found in much higher concentrations in the brain and other neuronal tissues [8–10]. This is probably to counter the increased likelihood of mischarging of D-amino acids. How, then, Gly-tRNA^Gly^ manages to escape misediting by DTD in these cells is an aspect that needs to be probed.

Another interesting aspect that emerges from this misediting by DTD is how certain isoacceptors of tRNA^Gly^ that have very poor affinity toward EF-Tu escape from DTD’s gratuitous activity. Glycine charged on these tRNAs is used for other metabolic purposes, such as cell wall synthesis in bacteria [56,57]. Probably, these tRNAs have evolved such negative determinants that prevent DTD from acting on them; alternatively, there may be some other cellular factor that is involved in providing protection to these species of Gly-tRNA^Gly^ against DTD. Hence, the aspect of protection of Gly-tRNA^Gly^ from DTD requires further, deeper inspection to gain a better understanding and appreciation of the dynamics or cross-talk that might be associated with the different molecules involved in this orchestra, i.e., Gly-tRNA^Gly^, EF-Tu, and DTD.

The other important aspect of DTD is its RNA-based catalysis. We previously showed that DTD-like fold (DTD and NTD) works in a side chain-independent mode to perform hydrolysis, and 2′-OH of A76 of tRNA was surmised to play a catalytic role [15,39,40]. Recently, we validated our hypothesis on the NTD system when we proved unequivocally the indispensible role of 2′-OH of tRNA in catalysis by NTDs [41]. We have now provided direct evidence of DTD being an RNA-based catalyst (Fig 7). DTD-like fold, therefore, brings us to a transitional scenario in evolution in which a primordial protein uses its peptide scaffold for proper binding/positioning of the substrate, thereby making conditions conducive for an RNA—tRNA in this case, which, interestingly, happens to be the substrate as well—to perform catalysis. Overall, the current work gives a new perspective on the fundamental aspect of how chirality-based proofreading is performed in bacteria and eukaryotes.

## Materials and Methods

### Cloning, Expression, and Protein Purification

EcDTD and PfDTD were cloned, expressed, and purified as described in [15]. DTD from *L*. *major* (LmDTD), *D*. *melanogaster* (DmDTD), and *D*. *rerio* (DrDTD) were cloned with C-terminal 6X-His tag into *Nco*I and *Xho*I sites of pET-28b (Novagen) expression vector. The proteins were overexpressed in *E*. *coli* BL21(DE3) expression strain and purified from the cell lysate using two-step purification protocol involving Ni-NTA affinity chromatography and size exclusion chromatography (SEC). The lysis buffer, used also for pre-equilibration of Ni-NTA column, contained 50 mM Tris–HCl pH 8.0, 150 mM NaCl, and 10 mM imidazole. After loading the Ni-NTA column with the cell lysate, the column was washed successively with wash buffers containing 50 mM Tris–HCl pH 8.0, 300 mM NaCl, and 30 mM imidazole, and 50 mM Tris–HCl pH 8.0, 150 mM NaCl, and 50 mM imidazole. Protein was eluted with elution buffer containing 50 mM Tris–HCl pH 8.0, 150 mM NaCl, and 250 mM imidazole. Fractions containing the protein were pooled, concentrated, and subjected to further purification with SEC (Superdex-75) in buffer containing 100 mM Tris–HCl pH 7.5 and 200 mM NaCl. Finally, the fractions containing the purified protein were pooled, concentrated, and mixed thoroughly with equal volume of 100% glycerol prior to aliquoting and storing at -30°C for future use.

### Co-crystallisation of PfDTD with Gly3AA

Purified protein was premixed with Gly3AA (Jena Biosciences, Germany) in a molar ratio of 1:20 and incubated overnight at 4°C. The protein–ligand premix was screened for initial crystallisation at 4°C and 20°C with Index, and Crystal screen 1 and 2 (Hampton Research, Aliso Viejo, CA), and JBS classic (Jena Biosciences) in sitting-drop setups using 96-well plates from MRC. Drops containing 1 μl protein–ligand premix and 1 μl reservoir solution were set using Mosquito crystallization robot (TTP LabTech, UK). Hits from the screen were further optimised using hanging-drop vapour diffusion method in 24-well Iwaki plates. Diffraction-quality crystals were obtained in 0.1 M HEPES pH 7.5, 0.4 M NaCl, 28% PEG3350.

### X-ray Diffraction Data Collection, Data Processing, and Structure Solution

X-ray diffraction data were collected at the in-house X-ray facility using FR-E+ SuperBright X-ray generator from Rigaku equipped with VariMax HF optic and R-AXIS IV++ image plate detector. Data processing was done using HKL2000 [58], while structure solution by molecular replacement was done using MOLREP-AUTO MR from the CCP4 suite [59] with PfDTD apo structure (PDB id: 3KNF) as the search model. The structure was refined using CNS [60] and REFMAC [61]. Model building was performed using COOT [62]. Restraints for ligand refinement were obtained from PRODRG server [63]. PROCHECK [64] and PyMOL Molecular Graphics System, Version 1.7.6.0 Schrödinger, LLC were used for structure validation and figure generation, respectively. The structure has been deposited in the Protein Data Bank with the accession code 5J61. Catalytic chamber volumes were calculated using the high-performance computational capabilities of the Helix Systems at the National Institutes of Health, Bethesda, MD (https://hpcwebapps.cit.nih.gov/structbio/). Structure-based multiple sequence alignment was done with T-Coffee web server using Expresso mode [65]. The figure depicting sequence alignment was prepared using ESPript 3.0 [66].

### Biochemical Assays

The assay conditions of EcDTD and PfDTD with various substrates have been described in [15]. A representative thin-layer chromatographic run is given in S8 Fig. *E*. *coli* tRNA^Gly^ was charged with glycine in a reaction mix containing 100 mM HEPES pH 7.2, 30 mM KCl, 10 mM MgCl_2_, 2 mM ATP, 50 mM glycine, 1 μM tRNA^Gly^, and 2.2 μM *T*. *thermophilus* glycyl-tRNA synthetase (UniProt ID: P56206) incubated at 37°C for 15 min. *E*. *coli* L-Ala-tRNA^Ala^ was generated by the protocol given in [25]. DTD deacylation kinetics on Gly-tRNA^Gly^ was performed following the method described in [25] and [27].

EF-Tu activation was done by incubating 10 μM *T*. *thermophilus* EF-Tu in a solution containing 50 mM HEPES pH 7.2, 20 mM MgCl_2_, 250 mM NH_4_Cl, 5 mM DTT, 2 mM GTP, 3 mM phosphoenol pyruvate, and 50 μg ml^-1^ pyruvate kinase at 4°C for 3 h. Competition (deacylation) assays were performed in a solution of 100 mM HEPES pH 7.2 and 2.5 mM DTT containing 2 μM activated EF-Tu, 200 nM Gly-tRNA^Gly^ and varying concentrations of EcDTD.

3′-end modifications of *E*. *coli* tRNA^Gly^ were generated using in vitro-transcribed tRNA^Gly^-CC lacking the terminal adenosine. Ten μM tRNA^Gly^-CC was incubated with [α-^32^P]-2′-dATP, 2.6 μM CCA-adding enzyme (UniProt ID: P06961), 50 mM Tris pH 7.6, 20 mM MgCl_2_, 0.5 mM DTT, and 0.6 mM CTP at 37°C for 4 h to generate tRNA^Gly^(2′-dA76). tRNA^Gly^(2′-FdA76) was similarly produced using 400 μM 2′-FdATP (Jena Biosciences) instead of [α-^32^P]-2′-dATP; however, tRNA^Gly^-CC used for making tRNA^Gly^(2′-FdA76) was body-labeled by performing in vitro transcription with [α-^32^P]-UTP. Aminoacyltion of the 3′-end modified tRNAs with glycine was done using the conditions given for wild-type tRNA charging. Time-point–based deacylation assays with Gly-tRNA^Gly^(2′-dA76) were performed using thin-layer chromatography–based method, whereas those with Gly-tRNA^Gly^(2′-FdA76) were done using acid urea polyacrylamide gel electrophoresis [67]. Each point on the deacylation curves represents the mean of three independent readings; the error bars represent one standard deviation from the mean.

### Cell Toxicity Assays

Cell toxicity assays were done with *E*. *coli* K12Δ*dtd*::Kan (available from Keio collection) and BL21(DE3) strains, which were transformed with EcDTD cloned in pTrc99 and pET-28(b) vectors, respectively, or with PfDTD cloned in pTrc99 and pET-21(b) vectors, respectively. As controls, the cells were also transformed with the corresponding empty vectors. In the case of LB–agar plate-based assay, a primary culture was grown in LB medium at 37°C until the OD_600_ reached 0.6. Subsequetly, six 10-fold serial dilutions were made in phosphate-buffered saline (PBS), and 3 μL of each was spotted on LB–agar plates supplemented with 0 or 1 mM IPTG in the case of EcDTD, or with 0 or 0.1 mM IPTG in the case of PfDTD. The plates were incubated at 37°C for 12–16 h. For liquid medium (LB)–based assay, a primary culture was grown as mentioned previously. Subsequently, 1% or 0.1% inoculum (for K12Δ*dtd*::Kan and BL21(DE3), respectively) was used to initiate 15 mL secondary culture supplemented with 0 or 0.1 mM IPTG and grown at 37°C. At different time points, OD_600_ was recorded. For assays with amino acid supplementation, the required amount of amino acid (glycine or L-alanine) was added to the conditions of growth and DTD overexpression mentioned above. With EcDTD, toxicity was not observed in growth curve, whereas supplementation assays did not show rescue from toxicity, probably because EcDTD overexpression has lesser toxic effect on the cell due to its lower biochemical activity compared to PfDTD.

### Transverse Relaxation Optimized NMR Spectroscopy

Bruker 600 MHz NMR spectrometer equipped with triple resonance cryoprobe (Bruker, Billerica, MA) was used for carrying out 2D ^15^N-^1^H TROSY experiments. Two hundred μM U-^15^N-PfDTD (C-terminal His-tagged) in 50 mM HEPES pH 7.0, 50 mM NaCl was titrated with each of D-Ala3AA, L-Ala3AA, and Gly3AA at protein-to-ligand molar ratios of 1:0, 1:5, 1:10, and 1:15. Data processing and figure preparation were done using Sparky.

### Mass Spectrometry Experiments

Deacylation of D-Tyr-tRNA^Tyr^ (approximately 20–40 μM) was done with PfDTD (approximately 5 μM) in approximately 40 μl H_2_O^18^ or H_2_O^16^ at 37°C for 1 h. The products were then passed through 10 kDa–cutoff membrane, and the flow-through, which contained the amino acid, was analysed using mass spectrometry. Electrospray ionisation (ESI) mass spectra were recorded using Exactive Orbitrap mass spectrometer (Thermo Scientific, US) in negative ion mode. Data were acquired using X calibur software (Thermo Scientific). The source conditions maintained were: sheath gas (N_2_) pressure, 25 psi; aux. gas pressure, 5 psi; capillary temperature, 200°C; capillary voltage, -50.0 V; tube lens offset voltage, -50 V; skimmer voltage, -40 V; vaporizer temperature, 250°C. Scanning parameters were: higher energy collision-induced dissociation (HCD) gas, off, resolution, enhanced micro scans, 5; lock masses, off AGC target, balanced and maximum injection time, 1,000 ms for a full-scan mass spectrum. All the sample solutions were infused into the ESI source at a flow rate of 10 μl min^-1^ by using the instrument’s syringe pump, and 20% methanol was used to improve spraying conditions. The spectra were recorded under identical experimental conditions for all the compounds with an average of 25–30 scans.

## Supporting Information

S1 Data Numerical data used in preparation of Figs 1B, 2C, 5A, 5B, 6B, 7A, S2, S4, S5B and S6B. (XLSX)

S1 Fig NMR spectroscopy–based binding studies of various post-transfer substrate analogs with PfDTD. Overlay of 2D ^15^N-^1^H TROSY obtained with 0.2 mM PfDTD (black) when titrated with 1 mM (green), 2 mM (red), and 3 mM (blue) of **(a)** D-Ala3AA, **(c)** L-Ala3AA, and (**e**) Gly3AA and their respective excerpts, **(b)**, **(d),** and **(f)**. **(g)** Overlay of 2D ^15^N-^1^H TROSY obtained with 0.2 mM PfDTD (black) when titrated with 3 mM of D-aspartyl-3′-aminoadenosine (green), D-seryl-3′-aminoadenosine (red), and D-Tyr3AA (blue). (**h**) Excerpts of the overlay in (**g**).
(TIF)

S2 Fig Biochemical activity of DTD on L-chiral substrate with higher concentrations of enzyme. Deacylation of L-Ala-tRNA^Ala^ by buffer (blue circle), 5 μM EcDTD (red triangle), 50 nM PfDTD (green square), and 5 μM PfDTD (brown diamond). The underlying data can be found in S1 Data.
(TIF)

S3 Fig Electron density maps of the ligand, Gly3AA. **(a)** 2*F*_*o*_*-F*_*c*_ maps contoured at 0.8σ for all the ligand molecules observed in the crystal structure. Since the electron density for the glycyl moiety was weaker than that for the adenine and ribose moieties, a lower contour level was used. It was observed that in most of the monomers, the α-NH_2_ group preferred the flipped orientation (i.e., the position in which Cβ is seen in D-Tyr3AA complexes), despite its inherent flexibility. The preference for the flipped orientation is probably due to a stronger interaction with the carbonyl oxygen of Pro150 than with the carbonyl oxygen of Gly149 in the original orientation. In a couple of monomers (chains C and F), the α-NH_2_ group could be placed in either orientation. In one monomer (chain H), there was no density for the α-NH_2_ group at all. Hence, in all the monomers, the α-NH_2_ group was placed and refined in the flipped orientation, as seen in the figure. **(b)** Stereo image of the electron density map (2*F*_*o*_*-F*_*c*_), contoured at 1.2σ, around the ligand-binding site showing Gly3AA (yellow) and protein residues of monomers E (orange) and F (green).
(TIF)

S4 Fig Atomic B-factor analysis of the ligand atoms. A plot of average atomic B-factor versus each ligand atom for all the ligand molecules observed in the crystal structure. Error bars indicate one standard deviation from the mean. Higher B-factor values indicate more flexibility. The underlying data can be found in S1 Data.
(TIF)

S5 Fig Mutational analysis of adenine-binding site. **(a)** Stereoscopic view of the adenine-binding site (stick representation) depicting the mutants (sphere representation). **(b)** Deacylation of Gly-tRNA^Gly^ by buffer (blue circle), EcDTD wild-type or PfDTD wild-type (green triangle), EcDTD A102F or PfDTD A112F (red square), and EcDTD F125A or PfDTD F137A (brown diamond). EcDTD was used at 50 nM, whereas PfDTD was used at 500 pM concentration in the assays. Error bars indicate one standard deviation from the mean. The underlying data of panel **(b)** can be found in S1 Data.
(TIF)

S6 Fig Growth defect/retardation due to DTD overexpression. **(a)** Growth of *E*. *coli* BL21(DE3) transformed with EcDTD or empty vector pET-28(b) (top panel), or with PfDTD or empty vector pET-21(b) (bottom panel) on LB–agar plates supplemented with 0 mM IPTG (left panel) or with 0.1 mM IPTG (right panel). **(b)** Growth curve of *E*. *coli* BL21(DE3) transformed with empty vector pET-21(b) and induced with 0 mM IPTG (blue circle), PfDTD and induced with 0 mM IPTG (red square), empty vector pET-21(b) and induced with 0.1 mM IPTG (green triangle), and PfDTD and induced with 0.1 mM IPTG (brown diamond). Error bars indicate one standard deviation from the mean. The underlying data of panel **(b)** can be found in S1 Data.
(TIF)

S7 Fig Mass spectrometry analysis of the deacylation product (amino acid). Analysis of the deacylation product (amino acid) after carrying out biochemical assay with PfDTD and D-Tyr-tRNA^Tyr^ in H_2_O^16^ **(a)** and H_2_O^18^ **(b)**. The peak for D-tyrosine corresponds to m/z value of 180.07 for reaction done in H_2_O^16^, and 182.08 for reaction performed in H_2_O^18^.
(TIF)

S8 Fig Representative thin-layer chromatographic run after deacylation assay. Time-point–based deacylation assay of Gly-tRNA^Gly^ and L-Ala-tRNA^Ala^ by EcDTD (50 nM and 5 μM, respectively) showing distinct bands for Gly-AMP and AMP, or L-Ala-AMP and AMP.
(TIF)

S1 Table Kinetic constants of EcDTD. (DOCX)

S2 Table Interaction distances of the glycyl moiety of Gly3AA from the protein atoms. (DOCX)

S3 Table Volume analysis of the active site pocket of DTD. (DOCX)

## Abbreviations

2′-dA76: 2′-deoxyadenosine 76 at 3’-terminal of tRNA
2′-FdA76: 2′-fluoro-2′-deoxyadenosine 76 at 3′-terminal of tRNA
A76: adenosine 76 at 3′-terminal of tRNA
aaRS: aminoacyl-tRNA synthetase
D-Ala3AA: D-alanyl-3′-aminoadenosine
DmDTD: DTD from *Drosophila melanogaster*
DrDTD: DTD from *Danio rerio*
DTD: D-aminoacyl-tRNA deacylase
D-Tyr3AA: D-tyrosyl-3′-aminoadenosine
EcDTD: DTD from *Escherichia coli*
EF-Tu: elongation factor thermo unstable
Gly3AA: glycyl-3′-aminoadenosine
L-Ala3AA: L-alanyl-3′-aminoadenosine
LmDTD: DTD from *Leishmania major*
NMR: nuclear magnetic resonance
NTD: N-terminal editing domain of archaeal ThrRS
PabNTD: NTD from *Pyrococcus abyssi*
PfDTD: DTD from *Plasmodium falciparum*
ThrRS: threonyl-tRNA synthetase
