# Supplementary figures and images for: Elongation Factor Tu Prevents Misediting of Gly-tRNA(Gly) Caused by the Design Behind the Chiral Proofreading Site of D-Aminoacyl-tRNA Deacylase
Source: PLoS Biol. 2016 May 25;14(5):e1002465. doi: 10.1371/journal.pbio.1002465 (PMC4880308; doi:10.1371/journal.pbio.1002465)

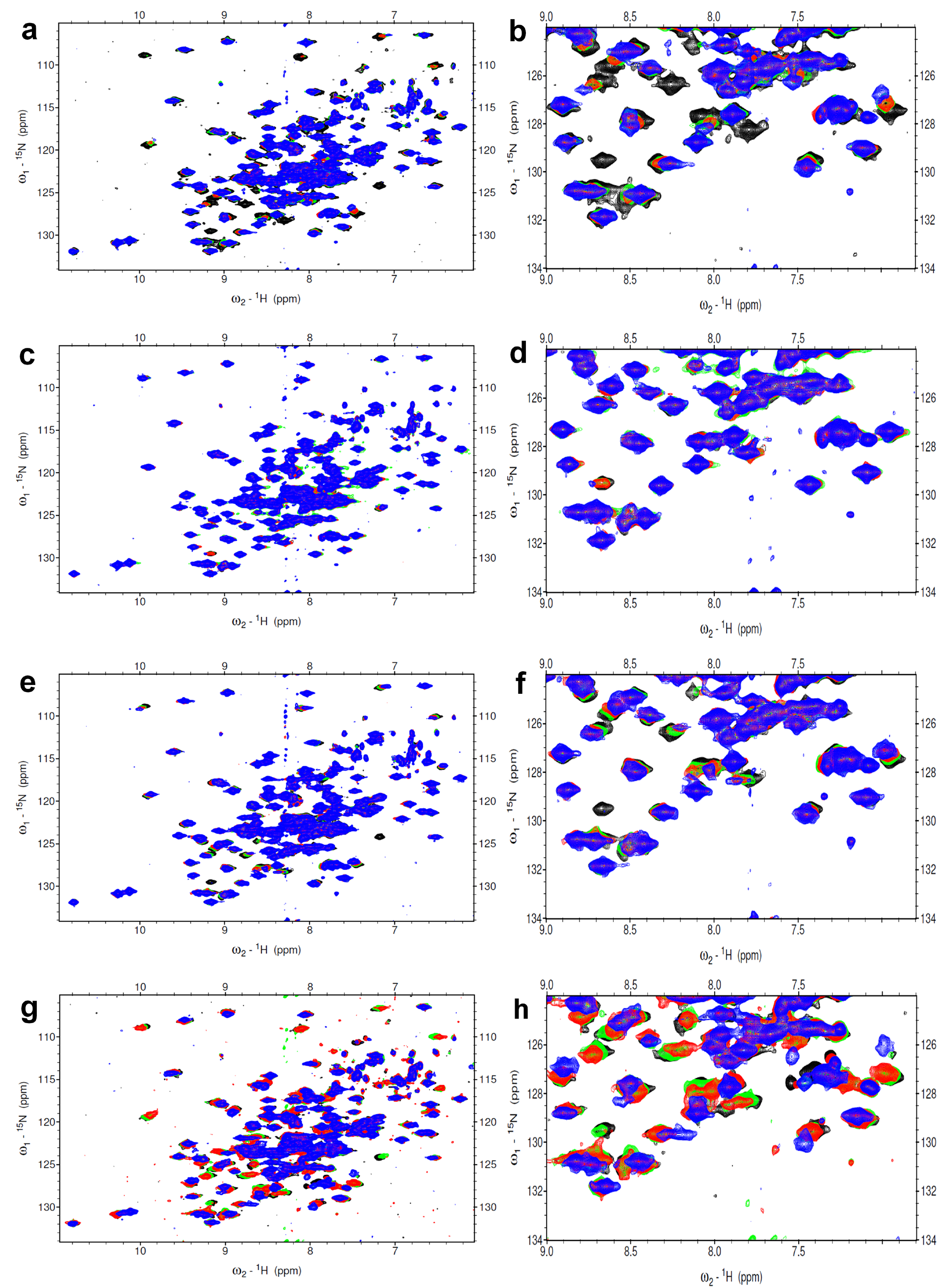

Supplement: S1 Fig — Overlay of 2D 15N-1H TROSY obtained with 0.2 mM PfDTD (black) when titrated with 1 mM (green), 2 mM (red), and 3 mM (blue) of (a) D-Ala3AA, (c) L-Ala3AA, and (e) Gly3AA and their respective excerpts, (b), (d), and (f). (g) Overlay of 2D 15N-1H TROSY obtained with 0.2 mM PfDTD (black) when titrated with 3 mM of D-aspartyl-3′-aminoadenosine (green), D-seryl-3′-aminoadenosine (red), and D-Tyr3AA (blue). (h) Excerpts of the overlay in (g). (TIF) [file pbio.1002465.s002.tif]

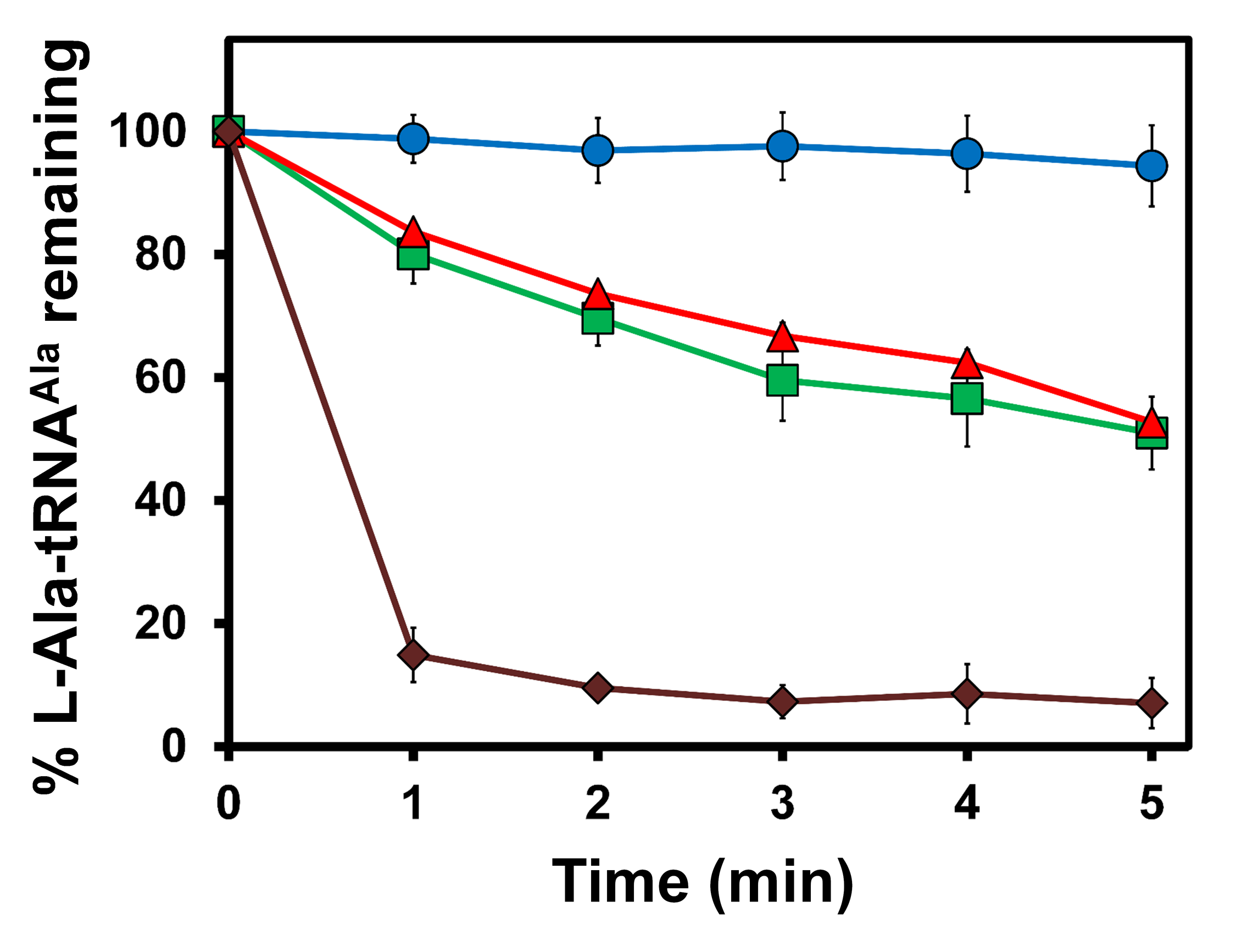

Supplement: S2 Fig — Deacylation of L-Ala-tRNAAla by buffer (blue circle), 5 μM EcDTD (red triangle), 50 nM PfDTD (green square), and 5 μM PfDTD (brown diamond). The underlying data can be found in S1 Data. (TIF) [file pbio.1002465.s003.tif]

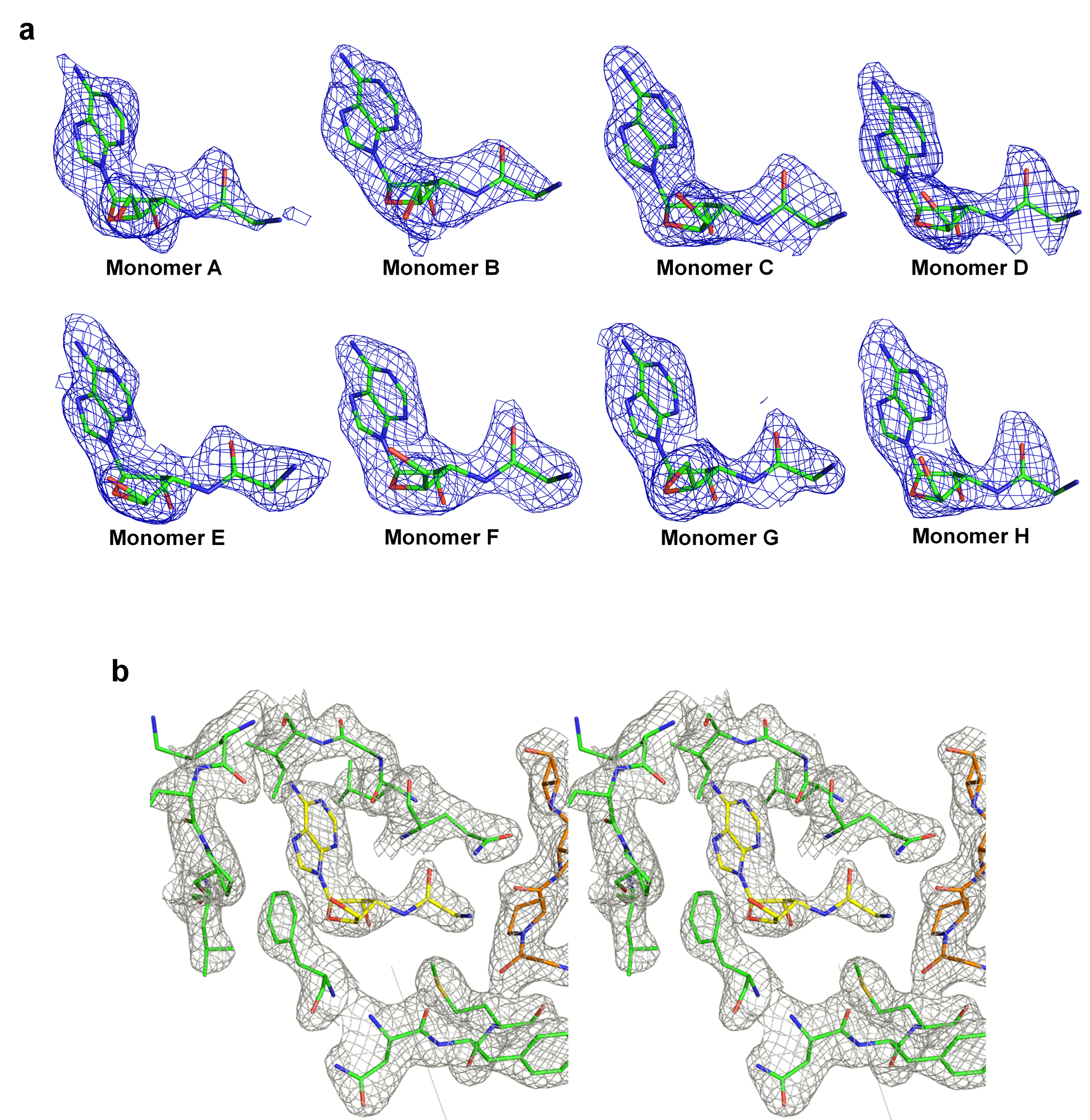

Supplement: S3 Fig — (a) 2Fo-Fc maps contoured at 0.8σ for all the ligand molecules observed in the crystal structure. Since the electron density for the glycyl moiety was weaker than that for the adenine and ribose moieties, a lower contour level was used. It was observed that in most of the monomers, the α-NH2 group preferred the flipped orientation (i.e., the position in which Cβ is seen in D-Tyr3AA complexes), despite its inherent flexibility. The preference for the flipped orientation is probably due to a stronger interaction with the carbonyl oxygen of Pro150 than with the carbonyl oxygen of Gly149 in the original orientation. In a couple of monomers (chains C and F), the α-NH2 group could be placed in either orientation. In one monomer (chain H), there was no density for the α-NH2 group at all. Hence, in all the monomers, the α-NH2 group was placed and refined in the flipped orientation, as seen in the figure. (b) Stereo image of the electron density map (2Fo-Fc), contoured at 1.2σ, around the ligand-binding site showing Gly3AA (yellow) and protein residues of monomers E (orange) and F (green). (TIF) [file pbio.1002465.s004.tif]

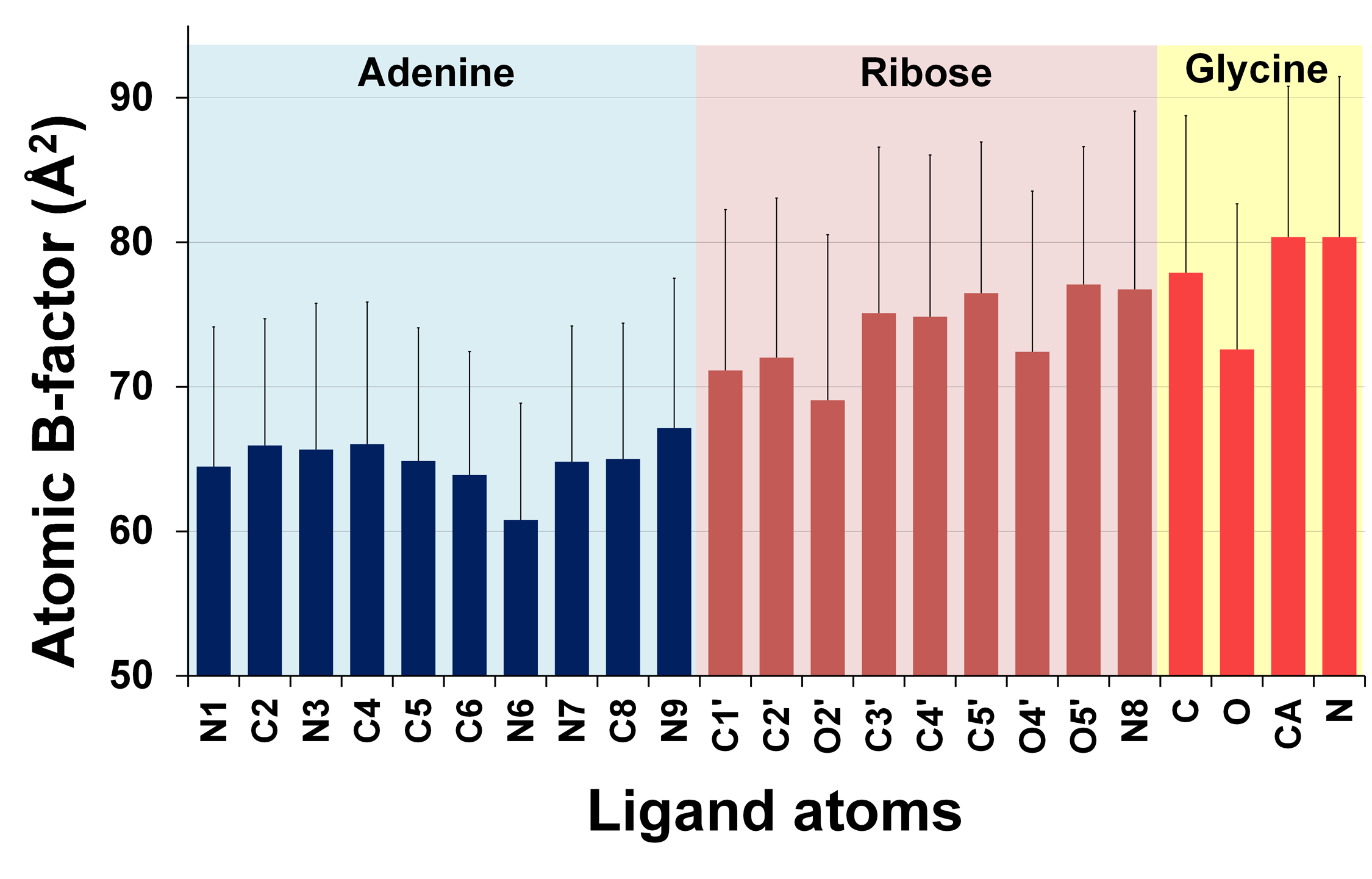

Supplement: S4 Fig — A plot of average atomic B-factor versus each ligand atom for all the ligand molecules observed in the crystal structure. Error bars indicate one standard deviation from the mean. Higher B-factor values indicate more flexibility. The underlying data can be found in S1 Data. (TIF) [file pbio.1002465.s005.tif]

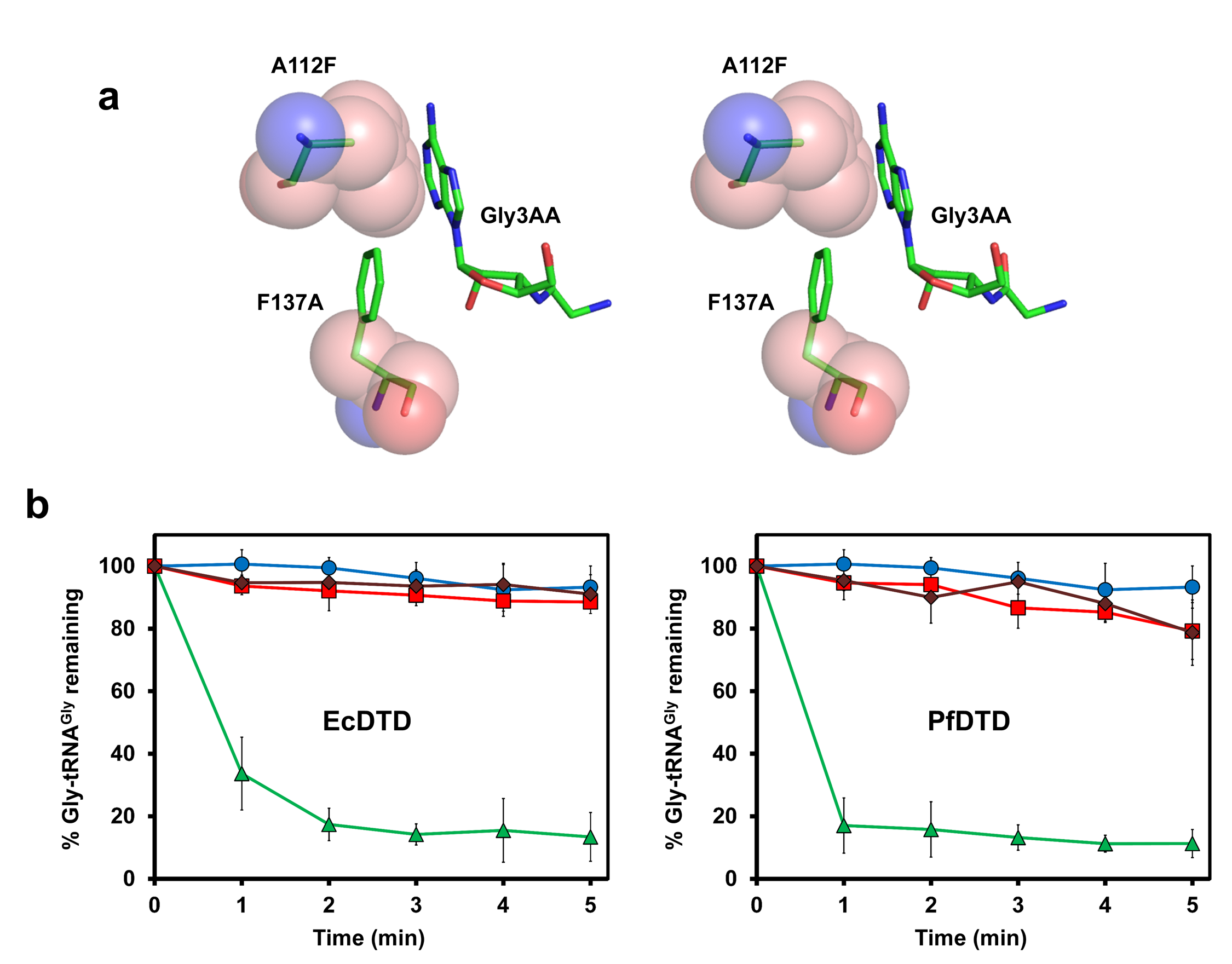

Supplement: S5 Fig — (a) Stereoscopic view of the adenine-binding site (stick representation) depicting the mutants (sphere representation). (b) Deacylation of Gly-tRNAGly by buffer (blue circle), EcDTD wild-type or PfDTD wild-type (green triangle), EcDTD A102F or PfDTD A112F (red square), and EcDTD F125A or PfDTD F137A (brown diamond). EcDTD was used at 50 nM, whereas PfDTD was used at 500 pM concentration in the assays. Error bars indicate one standard deviation from the mean. The underlying data of panel (b) can be found in S1 Data. (TIF) [file pbio.1002465.s006.tif]

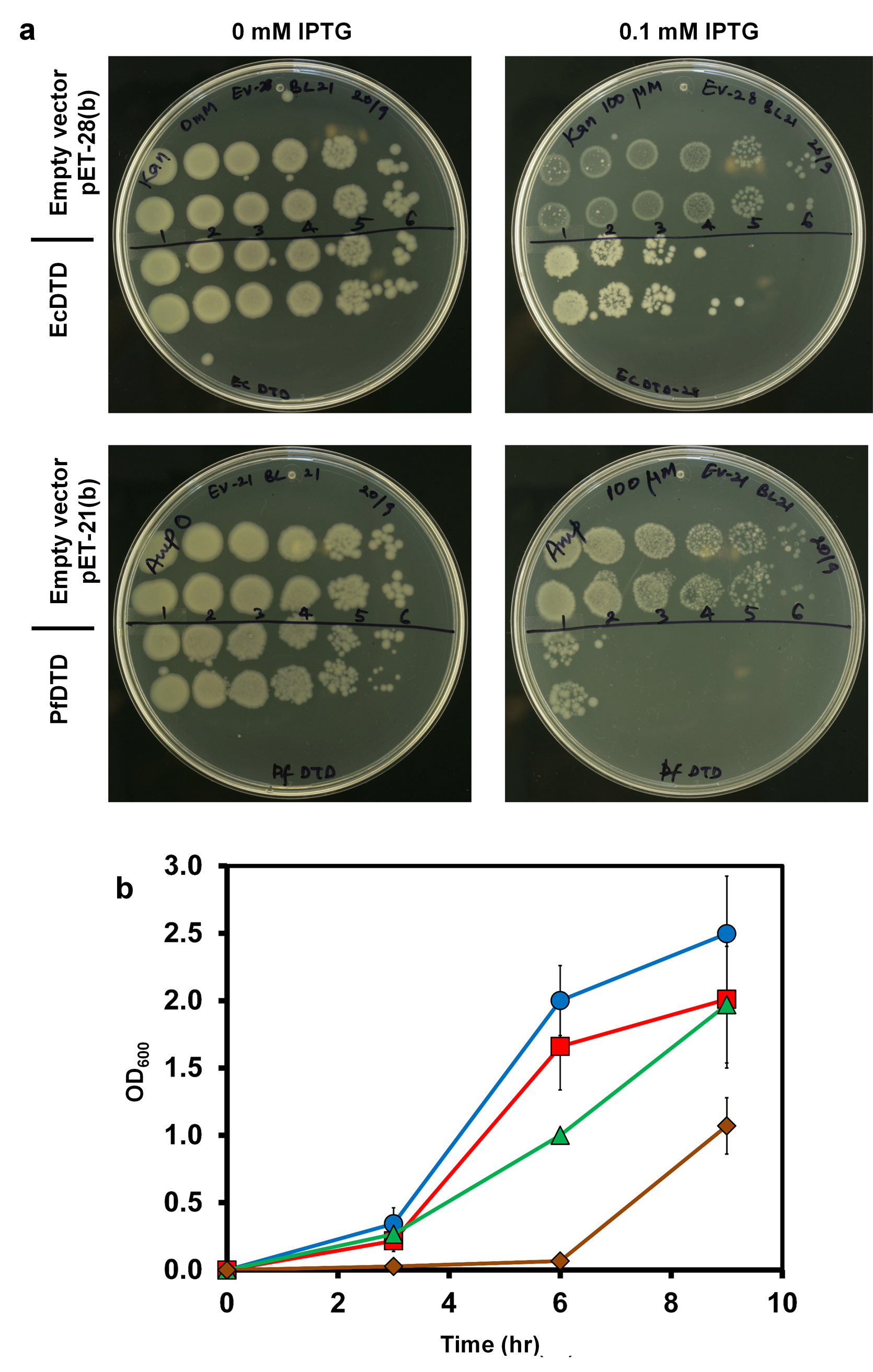

Supplement: S6 Fig — (a) Growth of E. coli BL21(DE3) transformed with EcDTD or empty vector pET-28(b) (top panel), or with PfDTD or empty vector pET-21(b) (bottom panel) on LB–agar plates supplemented with 0 mM IPTG (left panel) or with 0.1 mM IPTG (right panel). (b) Growth curve of E. coli BL21(DE3) transformed with empty vector pET-21(b) and induced with 0 mM IPTG (blue circle), PfDTD and induced with 0 mM IPTG (red square), empty vector pET-21(b) and induced with 0.1 mM IPTG (green triangle), and PfDTD and induced with 0.1 mM IPTG (brown diamond). Error bars indicate one standard deviation from the mean. The underlying data of panel (b) can be found in S1 Data. (TIF) [file pbio.1002465.s007.tif]

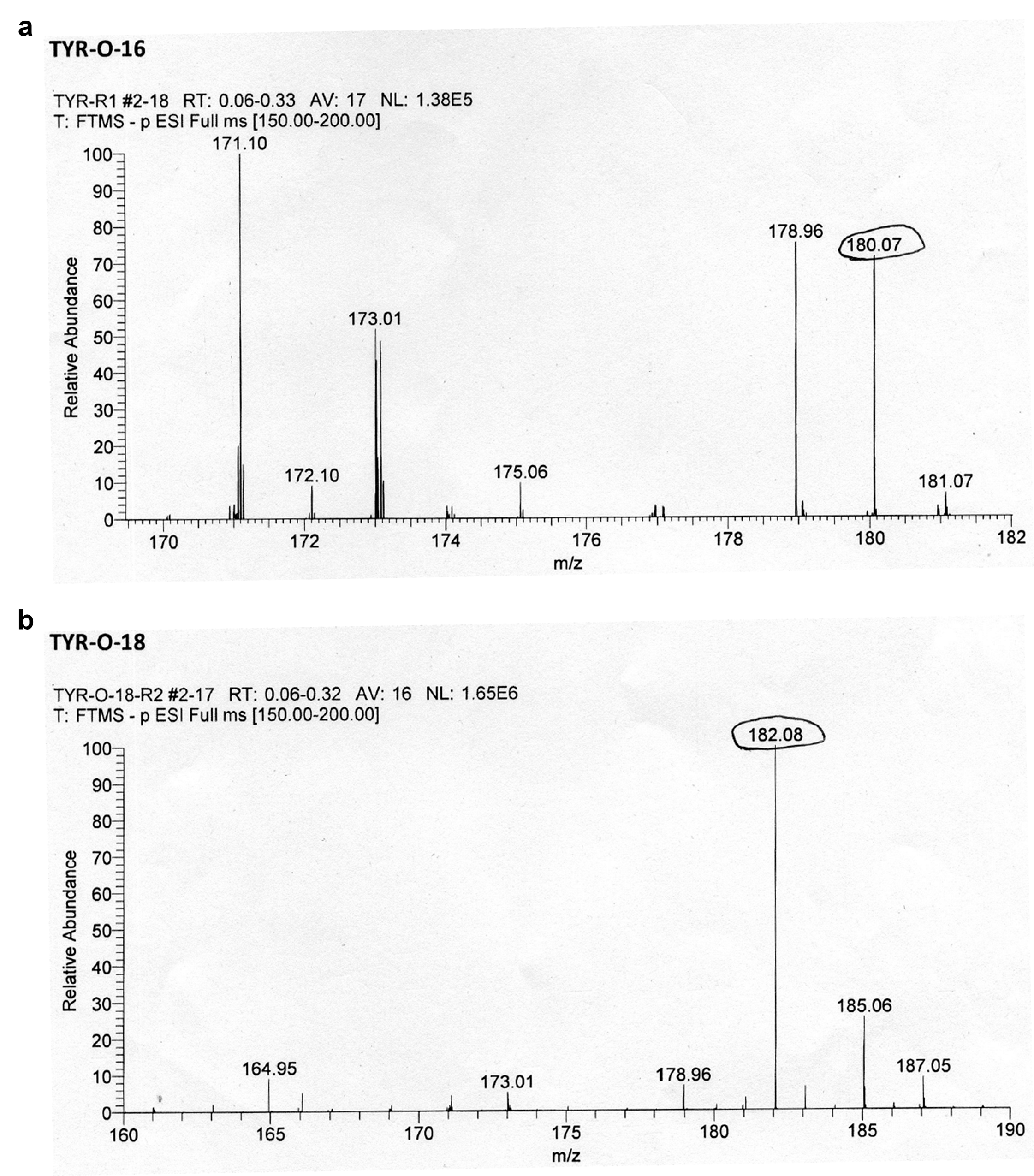

Supplement: S7 Fig — Analysis of the deacylation product (amino acid) after carrying out biochemical assay with PfDTD and D-Tyr-tRNATyr in H2O16 (a) and H2O18 (b). The peak for D-tyrosine corresponds to m/z value of 180.07 for reaction done in H2O16, and 182.08 for reaction performed in H2O18. (TIF) [file pbio.1002465.s008.tif]

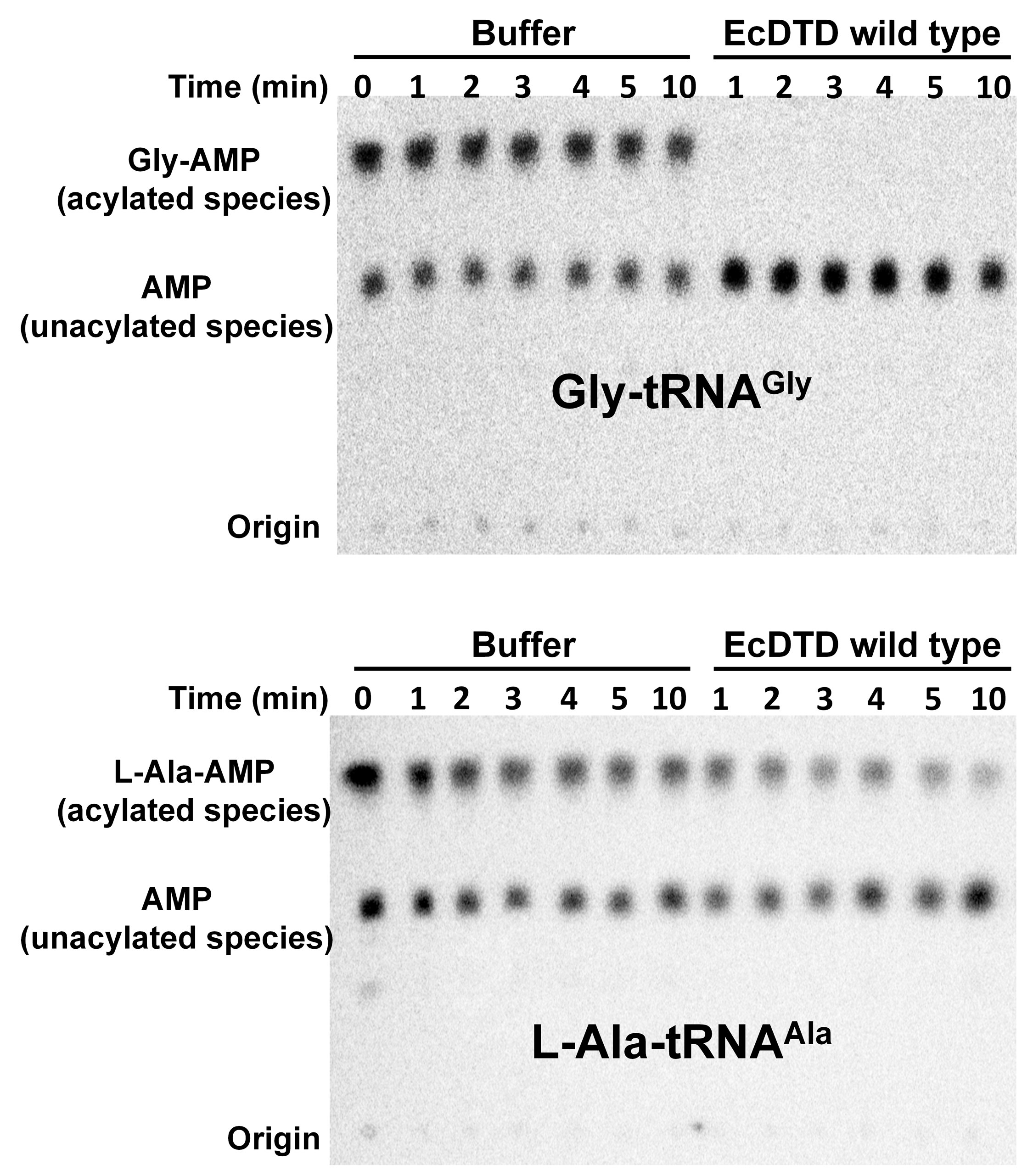

Supplement: S8 Fig — Time-point–based deacylation assay of Gly-tRNAGly and L-Ala-tRNAAla by EcDTD (50 nM and 5 μM, respectively) showing distinct bands for Gly-AMP and AMP, or L-Ala-AMP and AMP. (TIF) [file pbio.1002465.s009.tif]
