# Supplementary material for: Elongation Factor Tu Prevents Misediting of Gly-tRNA(Gly) Caused by the Design Behind the Chiral Proofreading Site of D-Aminoacyl-tRNA Deacylase
Source: PLoS Biol. 2016 May 25;14(5):e1002465. doi: 10.1371/journal.pbio.1002465 (PMC4880308; doi:10.1371/journal.pbio.1002465)
Supplement: S1 Table — (DOCX) [file pbio.1002465.s010.docx]

**S1 Table.** **Kinetic constants of EcDTD.**

| Substrate | k_cat_ (s^-1^) | K_m_ (µM) | k_cat_/K_m_ (µM^-1^ s^-1^) | Reference |
| --- | --- | --- | --- | --- |
| Gly-tRNA^Gly^ | **10** | **1** | **10** | **Our data** |
| D-Tyr-tRNA^Tyr^ | 6 | 1 | 6 | [13] |
| D-Trp-tRNA^Trp^ | - | - | 2.8 | [14] |
| D-Asp-tRNA^Asp^ | - | - | 12 | [14] |
