# Supplementary material for: Elongation Factor Tu Prevents Misediting of Gly-tRNA(Gly) Caused by the Design Behind the Chiral Proofreading Site of D-Aminoacyl-tRNA Deacylase
Source: PLoS Biol. 2016 May 25;14(5):e1002465. doi: 10.1371/journal.pbio.1002465 (PMC4880308; doi:10.1371/journal.pbio.1002465)
Supplement: S2 Table — (DOCX) [file pbio.1002465.s011.docx]

**S2 Table. Interaction distances of the glycyl moiety of Gly3AA from the protein atoms.**

| **Monomer** | **Ligand atom** | **Protein atom** | **Distance (Å)** |
| --- | --- | --- | --- |
| **A** | O | Phe89 N | 3.26 |
|  | O | Gln88 Nε | 3.14 |
|  | N | Pro150* O | 2.86 |
| **B** | O | Phe89 N | 3.34 |
|  | O | Gln88 Nε | 2.94 |
|  | N | Pro150* O | 3.22 |
| **C** | O | Phe89 N | 3.29 |
|  | O | Gln88 Nε | 3.08 |
|  | N | Pro150* O | 2.73 |
| **D** | O | Phe89 N | 3.15 |
|  | O | Gln88 Nε | 2.98 |
|  | N | Pro150* O | 2.88 |
| **E** | O | Phe89 N | 3.43 |
|  | O | Gln88 Nε | 3.02 |
|  | N | Pro150* O | 2.79 |
| **F** | O | Phe89 N | 3.32 |
|  | O | Gln88 Nε | 2.96 |
|  | N | Pro150* O | 3.26 |
| **G** | O | Phe89 N | 3.02 |
|  | O | Gln88 Nε | 3.15 |
|  | N | Pro150* O | 3.17 |
| **H** | O | Phe89 N | 3.48 |
|  | O | Gln88 Nε | 3.13 |
|  | N | Pro150* O | 3.07 |

* Residue from the dimeric counterpart
