# Supplementary material for: Elongation Factor Tu Prevents Misediting of Gly-tRNA(Gly) Caused by the Design Behind the Chiral Proofreading Site of D-Aminoacyl-tRNA Deacylase
Source: PLoS Biol. 2016 May 25;14(5):e1002465. doi: 10.1371/journal.pbio.1002465 (PMC4880308; doi:10.1371/journal.pbio.1002465)
Supplement: S3 Table — (DOCX) [file pbio.1002465.s012.docx]

**S3 Table. Volume analysis of the active site pocket of DTD.**

| **Crystal structure (PDB id)** | **Monomer** | ***Voronoi volume (Å^3^)** |
| --- | --- | --- |
| PabNTD+L-Thr3AA (3PD3) | A | 20.1 |
|  | B | 19.4 |
| PabNTD+L-Ser3AA (3PD2) | A | 25.2 |
|  | B | 22.9 |
| PfDTD+Gly3AA (present structure) | A | 30.7 |
|  | B | 29.2 |
|  | C | 25.1 |
|  | D | 23.8 |
|  | E | 30.5 |
|  | F | 27.3 |
|  | G | 25.8 |
|  | H | 25.4 |
| PfDTD+D-Tyr3AA (4NBI) | A | 24.7 |
|  | B | 23.2 |
| PfDTD+D-Tyr3AA (4NBJ) | A | 24.6 |
|  | B | 27.1 |
|  | C | 27.7 |
|  | D | 27.8 |
|  | E | 23.7 |
|  | F | 27.8 |
|  | G | 25.9 |
|  | H | 26.7 |

* The threshold volume for a water molecule (catalytic water) to be accommodated in the active site of DTD-like fold (i.e. DTD and its structural homolog such as PabNTD) is ~21 Å^3^. In PabNTD+L-Thr3AA, catalytic water is not observed, whereas in PabNTD+L-Ser3AA, catalytic water is observed. Complex structures of PfDTD have sufficient space to accommodate catalytic water, although the same has not been observed in any of the ligand-bound structures of DTD.
